# Multi-objective optimal synthesis of robust control systems for plants exhibiting non-minimum phase and integrating behaviour

**DOI:** 10.1038/s41598-025-24653-w

**Published:** 2025-11-19

**Authors:** M. Sai Neeharika, V. Shobhana, Nitish Katal

**Affiliations:** School of Electronics Engineering, Vellore Institute of Technology, Chennai, TN India

**Keywords:** Robust control, Optimal control, Multi-objective optimization, Sensitivity minimization, Energy science and technology, Engineering, Mathematics and computing

## Abstract

The classical controller design methods, often lead to sub-optimal performance, especially when implemented for plants exhibiting complex dynamics like integrals, non-minimum phase zeros, time-delays, etc.; and the controllers synthesised using classical methods can result in poor time domain characteristics, and limited robustness. Thus, it is essential to formulate the controller synthesis methods that improve stability, dynamic performance, and robustness. Proposed design explores the synthesis of optimal and robust controllers by posing the controller synthesis as a multi-objective optimization problem; wherein objectives of peak sensitivity, minimization of integral square error and control effort, along with phase margin penalty and delay margins are considered while formulating the objective function; followed by solving it by multi-objective genetic algorithm. Following the synthesis, a set of Pareto-optimal solutions is generated; to identify the ideal controller from these solutions, K-Means clustering is applied along with the determination of the utopia point for controller selection. The work is implemented for four systems like (a) integrating system, (b) position control of DC motor, (c) non-minimum phase hydropower system and (d) coupled tank systems. The proposed controller demonstrates significant quantitative improvement of performance metrics across all systems when compared to conventional methods. Additionally, Monte Carlo simulations for the robustness analysis are included to establish the superiority of the proposed method over the conventional.

## Introduction

In industrial control systems, synthesising both a robust and an optimal controller is extremely critical^1^. Classical control methods generally aim at achieving closed loop stability and try to meet basic performance requirements. However, these classical designs often lead to non-optimal solutions. In process control industries, it is essential to assure good compensation and regulation; as any variations in the process output because of noise or disturbances will directly impact the product’s quality and economy of the industry^2^. Certain industrial processes characterize and inherent complicated dynamics due to saturation, presence of poles at origin, and/or due to non-minimum phase characteristics. The integral systems can lead to sluggish or unstable behaviour due to accumulated errors^3^, whereas the non-minimum phase (NMP) systems are challenging due to their inverse response and reduced phase margin^4^. It is essential to address these issues to ensure reliability of these processes and this often requires sophisticated control strategies and careful controller tuning to balance performance and stability.

These issues in stability and performance can be tackled by implementing robust control techniques’; however, synthesis of robust control systems is inherently complex^5^. A control system is deemed robust only if it ensures stability and/or performance despite uncertainties’ such as parametric variations, nonlinearities, and unmodeled dynamics^5,6^. Achieving these objectives requires searching complex decision spaces, and synthesising controllers that are sufficiently robust and practically implementable. Furthermore, there it is essential to ensure simplicity in design and real-world implementation.

Robust control theory aims at synthesising a controller that is able to handle the uncertainties arising in a control system^7^. Sometimes, just having a robust controller does not assure optimality of the control, as in case of classical robust control methods a high order controller is obtained; thus, it becomes essential to ascertain the optimality of the robust control systems such that an optimal and robust response despite uncertainties and external disturbances; and the assurance of both optimality and robustness enhances the reliability and fault tolerance of the synthesised systems^8^.

In the existing literature, the nature-inspired algorithms (NIAs) have proven to be successful in solving complicated real-life problems, be it in operational research, finance, engineering, transportation systems, medical sciences, etc^9^. In control systems, NIAs have been widely used for the controller optimization; and have reported to offer better solutions when compared to convex optimization methods; as the controller synthesis is a multi-objective optimization problem (MOOP)^10^. In^11^, a comprehensive survey is presented on various PID controllers synthesis methods, wherein the emphasis is laid on methodologies for the optimal controller synthesis and self-tuning capabilities. In^12^, a decentralized $\:{\mathcal{H}}_{\infty\:}$ controller is proposed for coupled tank systems which also improves the disturbance rejection and reference tracking. In^13^, the optimal synthesis of the FOPID controllers is addressed using GA for the industrial liquid level systems; wherein it has been reported that the GA tuned FOPID controllers offers better time domain characteristics when compared to the classical methods like Ziegler Nichols. In^14^, the optimal design of QFT controllers for non-minimum phase (NMP) hydropower system considering the objectives of robustness and tracking; wherein it is found that the quantitative feedback theory (QFT) controllers offered robust response for parametrically uncertain plant. In^15^, multi-objective synthesis of controllers is carried out for the sequencing batch reactor in a water treatment plant; which offered better performance when compared with the classical methods. In^16^, a new robust control technique is reported for the design of FOPD controllers for integrating systems using multi-objective optimization along with experimental verification. In^17^, multi-objective design of PID controllers is proposed considering the objectives of peak sensitivity and tracking performance for higher order systems and time delay systems and the ideal Nash solution is chosen at multi-criteria decision making stage. In^18^ a two-degree-of-freedom modified smith predictor is proposed for the control of time-delay systems; architecture features a PID, PD an lag/lead compensator; and the controller gains have been found using pole placement to assure tracking and robustness. In^19^ multi-objective synthesis of PI controllers is considered for the speed control of BLDC motors in EVs considering the weighted objectives of ISE and energy consumption,.

It has also been reported in literature that, for the systems with complex dynamics, synthesizing multi-objective controllers is essential for balancing trade-off between conflicting objectives like stability, disturbance rejection, control effort minimization, and reference tracking^20^. Especially, for the plants that exhibit non-minimum phase (NMP) characteristics or integrating behaviour, achieving a balanced control performance can be particularly difficult. In^21^ a comparative study is presented amongst classical PID, IMC and GA optimal PID controllers the optimal load frequency control in NMP hydro power systems. Multi-objective synthesis enables the simultaneous fulfilment of the multiple performance criteria, thus allowing for the satisfying these objectives simultaneously^22^. Additionally, complex systems like non-minimum phase systems and integral systems exhibit inherently complicated dynamics; which can make their control challenging; as these characteristics poses challenge for the designed controller to consistently assure performance in the presence of uncertainties. For example, the non-minimum phase hydroelectric power systems, where load frequency control is vital, initial frequency dips following input changes complicate the stability assurance and responsive control; whereas in case of integrating plants, commonly found in applications like temperature or level control, can suffer from error accumulation over time, resulting in drift or oscillations; for example in systems like DC motors, variations in parameters can result in significant changes in response under different load conditions. Whereas, coupled tank systems can show unpredictable behaviour because the levels in the tanks are interdependent and the dynamics are coupled; for instance, a change in inflow to one tank can cause the level in another tank to drop^12^. So, the use of nature-inspired algorithms presents a promising solution to synthesise robust and optimal controllers for such class of systems.

To address these challenges, the present work formulates controller synthesis as a multi-objective optimization problem (MOOP), considering the objectives of peak sensitivity, minimization of integral square error (ISE) and control effort, along with phase margin penalty and delay margins are considered while formulating the objective function; followed by solving it by MOGA. After synthesis, Pareto optimal solutions (POS) are obtained; and K-Means clustering is applied to determine the utopia point, enabling the selection of the optimal controller. The work is implemented for four plants viz., integral system, position control of DC motor, non-minimum phase hydro power systems and coupled tank systems. The results have been compared with classical controller designed using Ziegler-Nichols, also with optimal controllers derived using GA considering the objective of minimization of ISE using genetic algorithm and the methods reported in existing literature; and current findings highlight superior performance exhibited by the proposed synthesised controllers, where they offer better closed loop tracking characteristics and robustness to parametric uncertainties. The robustness of the proposed controller is also verified and compared with the existing ones using Monte Carlo simulations.

The paper is organised as follows: Sect. 2 provides background on multi-objective controller synthesis and the considered plants; Sect. 3 presents the problem formulation; Sect. 4 details the obtained results; Sect. 5, highlights the procedure for selection of ideal solution from the POS using K-Means clustering; Sect. 6 compares the present design with existing methods; Sect. 7 validates the performance under parametric uncertainties’; Sect. 8 validates the robustness using Monte Carlo simulations and Sect. 9 discusses the findings and followed by conclusions.

## Background

Here the fundamentals of multi-objective controller synthesis, multi-objective genetic algorithm, and the dynamics of the plants considered for the evaluation is discussed.

### Multi-objective controller synthesis

Most of the industrial control systems require the attainment of one or more performance objectives. The controller synthesised using classical control theory might not be able to assure the attainment of all such required performance indices^23^. Along with that, these industrial control systems inherent complicated dynamics, because of non-minimum phase, integrating poles, time-delay etc., making the controller synthesis even more challenging. So, for such processes, the classical control theory will not be able to assure optimal performance as well as robustness. So, to address such conflicting objectives and assure optimality of the solution, the controller synthesis is posed as a MOOP; wherein various tracking and robustness performances can be attained simultaneously and Pareto optimal set (POS) of solutions can be obtained; and the control designer can choose the optimal controller as per the desired trade-off among design objectives^20^. Mathematically, multi-objective controller synthesis can be done by defining a MOOP which is presented by Eq. (1).

1 $$\ \begin{gathered} {\text{min }}J\left( x \right) = \left\{ {J_{1} \left( x \right), \ldots \:,\:J_{n} \left( x \right)}\} \right.\: \hfill \\ {\text{Subject}}\:{\text{to}}\:{\text{constraints}},\:g\left( x \right) \le \:0;\:h\left( x \right) = \:0;\underline {x }_{i}\le \:x_{i} \le \:\overline{{x_{i} }} \hfill \\ \end{gathered}$$

where $\:{x}_{i}$ is the decision vector in the search space; $\:J\left(x\right)$ is the objective vector; $\:g\left(x\right)$, $\:h\left(x\right)$ are the inequality and equality constraints respectively; and $\:\underset{\_}{{x}_{i}},\:\overline{{x}_{i}}$ are the lower and upper bounds on decision vector. This problem will yield a set of Pareto optimal solution set defined $\:{\varOmega\:}_{p}$.

### Plants

#### Integrating systems

An integrating plant is a dynamic system whose output is based on integral of input signal over time. The transfer function of an uncertain DC motor is given by Eq. (2).

2 $$\:G\left(s\right)=\frac{ka}{s\left(s+a\right)}=\frac{25}{s\left(s+5\right)}$$

where, $\:k$ and $\:a$ are uncertain parameters; with nominal value 5.

#### Position control of a DC motor

The transfer function of position control of a DC motor considered is given by Eq. (3). Where moment of inertia of rotor *J* is 3.2284 × 10^−6^ kg.m^2^, constant for motor viscous friction *b* is 3.5077 × 10^−6^ N.m.s, constant for EMF *K*_*b*_ is 0.0274 V/rad/sec, constant for motor torque *K*_*t*_ is 0.0274 N.m/Amp, resistance *R* is 4 Ohm and inductance *L* is 2.75 × 10^−6^H^24^.

3 $$\:G\left(s\right)=\frac{K}{s\left(\left(Js+b\right)\left(Ls+R\right)+{K}^{2}\right)}=\frac{0.0274}{s\left(8.878\times\:{10}^{-12}{s}^{2}+{1.291\times\:10}^{-5}s+0.0007648\right)}$$

#### Non minimum phase (NMP) hydropower plant

**Fig. 1:**
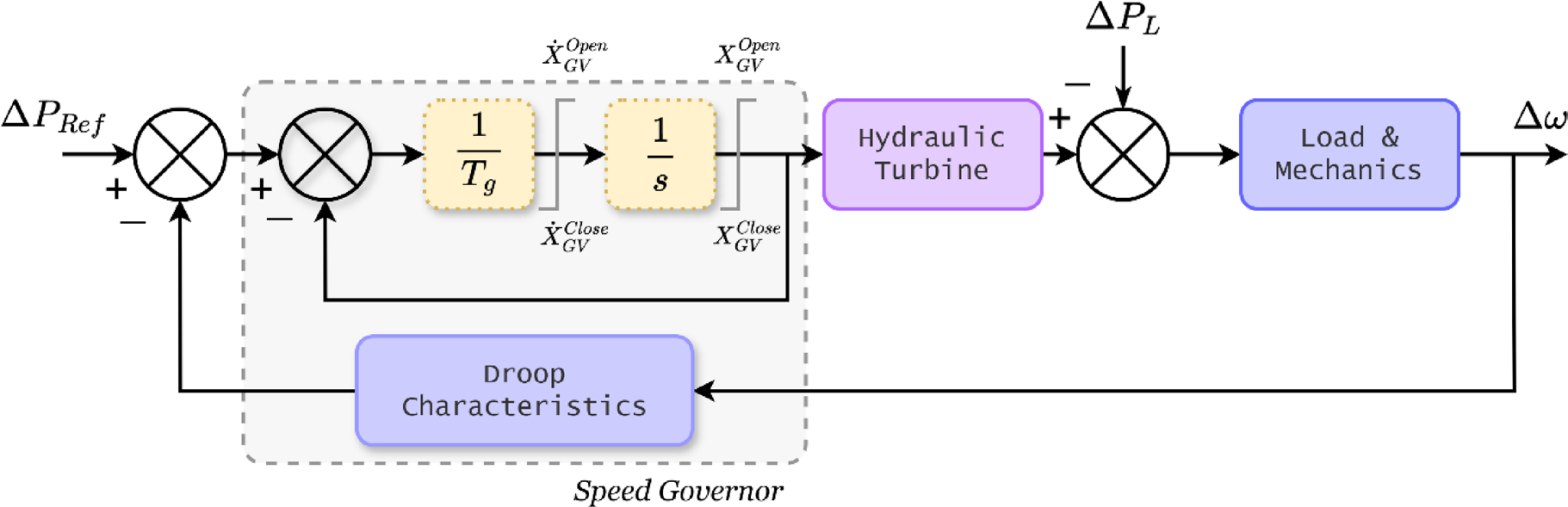
Block diagram of turbine, governor, load and machine.

In a power system, frequency stability is achieved through coordinated actions of turbo generators and their governors, which respond to load changes by adjusting turbine speed and water flow, as shown in Fig. 1. As shown in Eq. (4), the power system exhibits a right-half-plane zero, categorizing it as a non-minimum phase (NMP) system and thereby complicating controller design. NMP systems, unlike minimum phase systems, can become unstable even for bounded inputs, constraining control bandwidth and disturbance rejection^14^.

4 $$\:G\left(s\right)=\frac{\left(1-0.5\cdot\:s\right)}{\left(1+4\cdot\:s\right)\left(1+0.25\cdot\:s\right)\left(1+6\cdot\:s\right)}$$

#### Coupled tank system

**Fig. 2:**
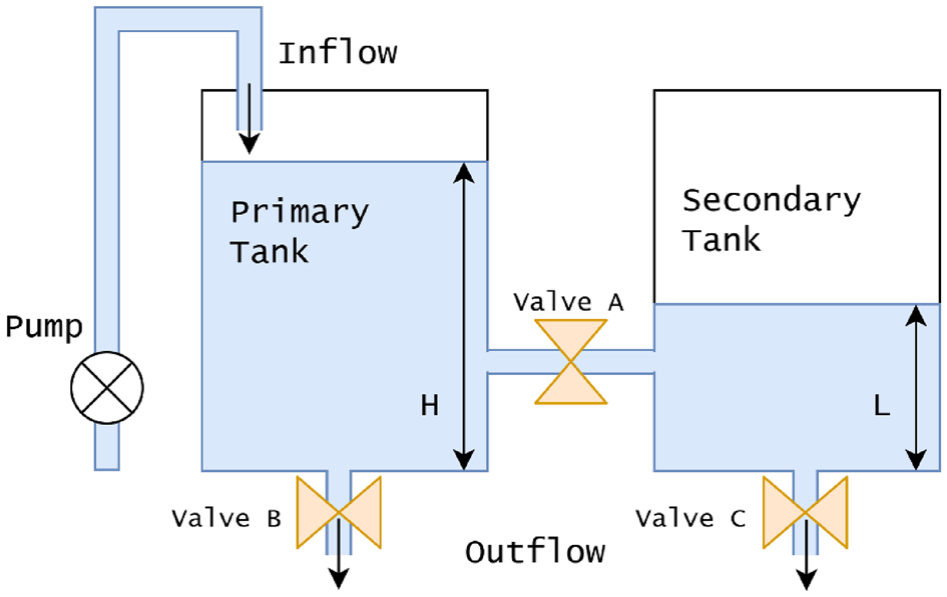
Schematic of a coupled tank system.

Coupled tank system is widely employed across industries like petrochemical, water treatment, etc. The accurate level regulation is essential, since the secondary tank’s concentration depends upon the primary tank’s flow. Figure 2 illustrates the system, and it’s transfer function is given in Eq. (5)^25^.

5 $$G(s)=\frac{{10.2}}{{500{s^2}+510s+1}}$$

## Multi-objective controller synthesis: problem formulation

As, most of the industrial processes requires the attainment of multiple time and frequency domain performance objectives and constraints; the attainment of such performances becomes challenging in case of processes with complex dynamics. Thus, it is essential to include these metrics in the controller synthesis problem, so that an optimal performance can be assured. So, to address this, the controller synthesis can be posed as an optimization problem, either as a weighted aggregate of functions or as based on generate-first choose-later approach; thus, an optimal solution with almost perfect trade-off between robustness and optimisation can be obtained, such that the optimal time domain tracking and robustness to parameter variation, uncertainties and noise can be dealt with. The present work takes in to consideration five objectives of (a) peak sensitivity minimization criterion, (b) control effort minimization, (c) minimization of integral square error, (d) delay margin and d) phase margin penalty.

### Peak sensitivity $\:{M}_{S}$

The first objective considered here is the minimization of peak sensitivity. The sensitivity function is used to describe the impact of the disturbance and the model uncertainties on closed loop response of system. Sensitivity function $\:S\left(s\right)$ is given by Eq. (6), the minimization of the peak sensitivity $\:{M}_{S}$ is given by Eq. (7) as:

6 $$\:S\left(s\right)=\frac{1}{1+G\left(s\right)K\left(s\right)}$$

7 $$\:{J}_{PS}=\text{min}\left\{\underset{\omega\:}{\text{max}}\left|S\left(j\omega\:\right)\right|\right\}$$

Peak sensitivity is used for the quantification of the worst-case amplification of the input disturbances and the unmodeled dynamics. In the case of the integrating systems, the presence of the low-frequency poles leads to a potential drift and high error accumulation, leading to an increase in the peak sensitivity. Also, in case of NMP systems, presence of right-hand side poles introduces the phase lag and initial inverse response, which might lead of the amplification of the disturbance if the peak sensitivity is high. Thus, the minimization of the peak sensitivity ensures disturbance rejection, improved damping and minimizes excessive overshoots.

### Minimization of control effort

The second objective, the minimization of the control effort, $\:u\left(t\right)$, aids in minimizing amount of control input or energy required to manipulate given plant and is given by Eq. (8). This aids in achieving desired closed loop performance and also to enhance the overall energy efficiency and reduce the load on the actuators.

8 $$\:{J}_{CE}={\int\:}_{0}^{T}u{\left(t\right)}^{2}dt\:$$

Typically, the high gain controller can aid in achieving faster tracking performances, but leads to larger control signals, potentially leading to actuator wear or saturation. Also, in case of the integrating plants, to counter the drift, they require persistent control; while in case of NMP systems a higher initial control effort is needed to counter the inverse dynamics. Thus, the minimization of the control effort will ensure an energy efficient control, and prevent actuator wear or saturation.

### Integral square error

The third objective is the minimization of the integral square error; which serves as a performance measure to evaluate how effectively a control system follows its desired output or setpoint over time. ISE is defined by integrating the square of the error signal $\:e\left(t\right)$. Mathematically, it can be expressed using Eq. (9):

9 $$\:{J}_{ISE}={\int\:}_{0}^{T}{\left(e\left(t\right)\right)}^{2}dt={\int\:}_{0}^{T}{\left(r\left(t\right)-y\left(t\right)\right)}^{2}dt$$

Where, $\:e\left(t\right)$ is error signal, $\:r\left(t\right)$ is reference signal or desired output and $\:y\left(t\right)$ is the actual output. The minimization of the ISE ensures the minimization of the large errors and sustained deviations over time. In case of the NMP systems, initial undershoots and inverse response significantly contributes to the ISE; while in case of the integrating plants the minimization of the ISE aids in minimizing the accumulation of the small steady-state offsets over time.

### Phase margin penalty

Phase margins ensure sufficient damping to avoid oscillatory behaviour in the control systems. In the case of integrating systems, which characterize low phase at low frequencies, making them prone to slow or oscillatory responses; likewise in case of NMP systems, the presence of RHP zeros minimizes achievable phase margins and results in limited fast control actions. Thus, the penalization of the low phase margins will aid in the synthesis of the controller that maintains adequate stability margins, and thus preventing oscillations. The objective for phase margin penalty is given by Eq. (10).

10 $$\:{J}_{PMP}=\text{max}(\text{0,45}^\circ\:-PM)$$

### Delay margin

The delay margin is used for the quantification of tolerance of closed loop system to delays. Both integrating and the NMP systems are highly sensitive to the time delays; and even the presence of small delays may destabilise the system. Thus, posing a penalty on the insufficient delay margin ensures that the controller maintains a robustness to delays. Mathematically is given in Eq. (11).

11 $$\:{J}_{DM}=\text{max}(0,D{M}_{MIN}-DM);\:\:\:\:\text{where},\:\:\:DM=\frac{1}{{\omega\:}_{180}}$$

Where, $\:{\omega\:}_{180}$ is frequency at which open loop phase crosses − 180°, and *DM*_*MIN*_ is the minimum acceptable delay margin (e.g., 0.2 s.)

In the present work, using the above mentioned objectives of peak sensitivity, minimization of control effort, the minimization of the integral square error, along with delay margin and phase margin penalty aids in formulating on objective function that ensures that the critical criterion of robustness is met, along with a closed loop system that has less control effort and also ensures good tracking performance; thus holistically satisfying the robustness, tracking and energy constraints and criterions. In this work, problem is framed as a MOOP, represented mathematically by the Eq. (12).

12 $$\:J\left(x\right)=\text{m}\text{i}\text{n}\:\{\:\:{J}_{PS},\:{J}_{CE},\:{J}_{ISE},{J}_{PMP},{J}_{DM}\}$$

**Fig. 3:**
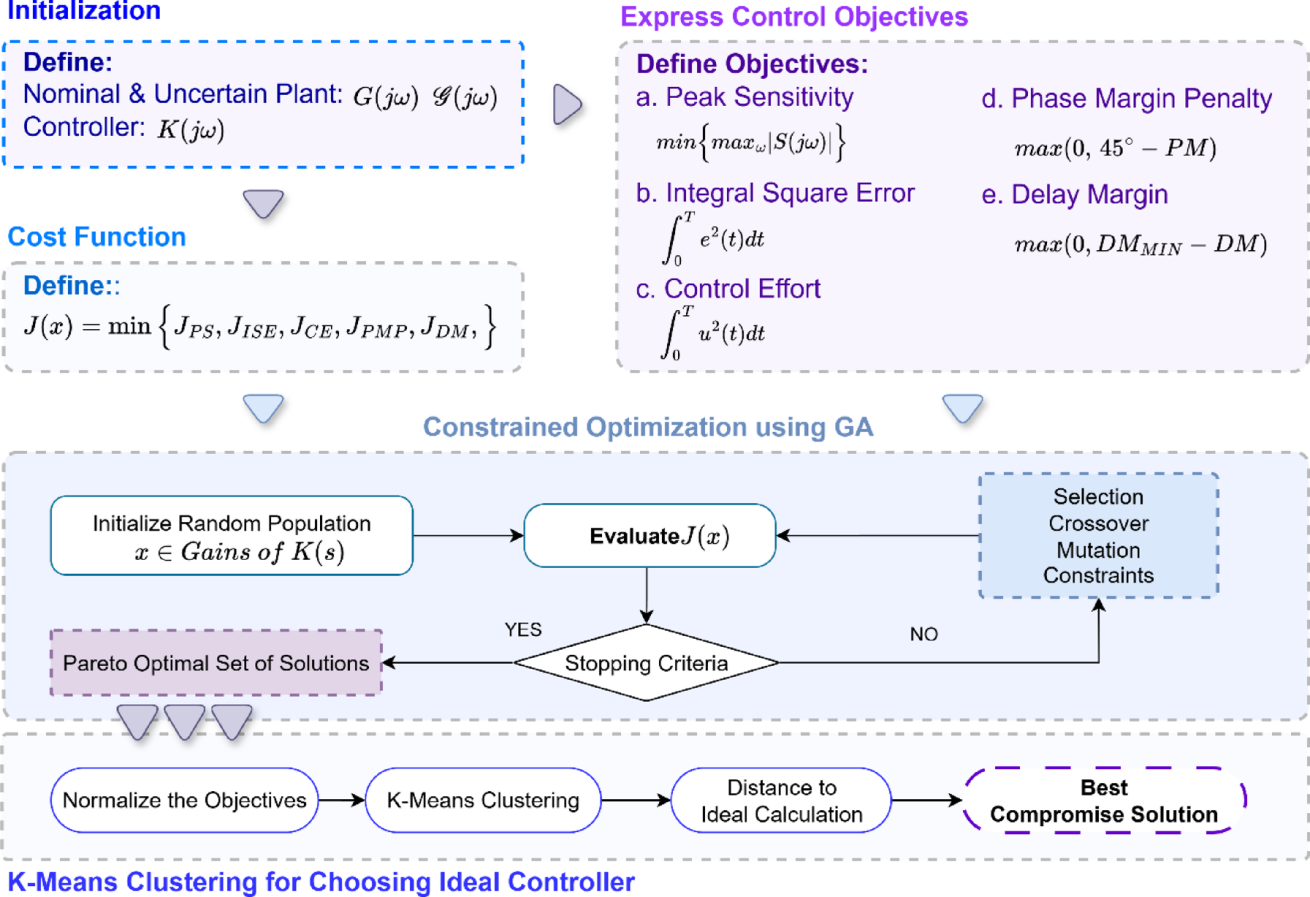
Block diagram of optimisation process

Subject to the bounds $\:\underset{\_}{{x}_{i}}\le\:x\le\:\overline{{x}_{i}},i=\left[1,\dots\:,n\right]$ and each $\:{x}_{i}\:$ has 4 decision variables corresponding to proportional, integral and derivative gains $\:{K}_{P}$, $\:{K}_{I}$, $\:{K}_{D}$ and $\:N$ (which first-order derivative filter time constant). The flowchart is illustrated in Fig. 3.

## Results

Here multi-objective synthesis of controllers using the above formulated multi-objective design problem for synthesising the PID controllers is discussed. The problem is solved using non-dominated sorted genetic algorithm – II (NSGA-II) which preserves elitism in the subsequent solutions; and returns a set of Pareto optimal solutions (POS).

### Plant 1: Integrating system

**Fig. 4:**
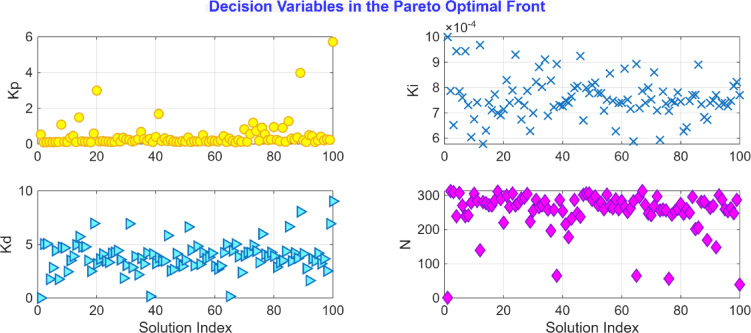
Controller parameters in the POS for integrating plant

**Fig. 5:**
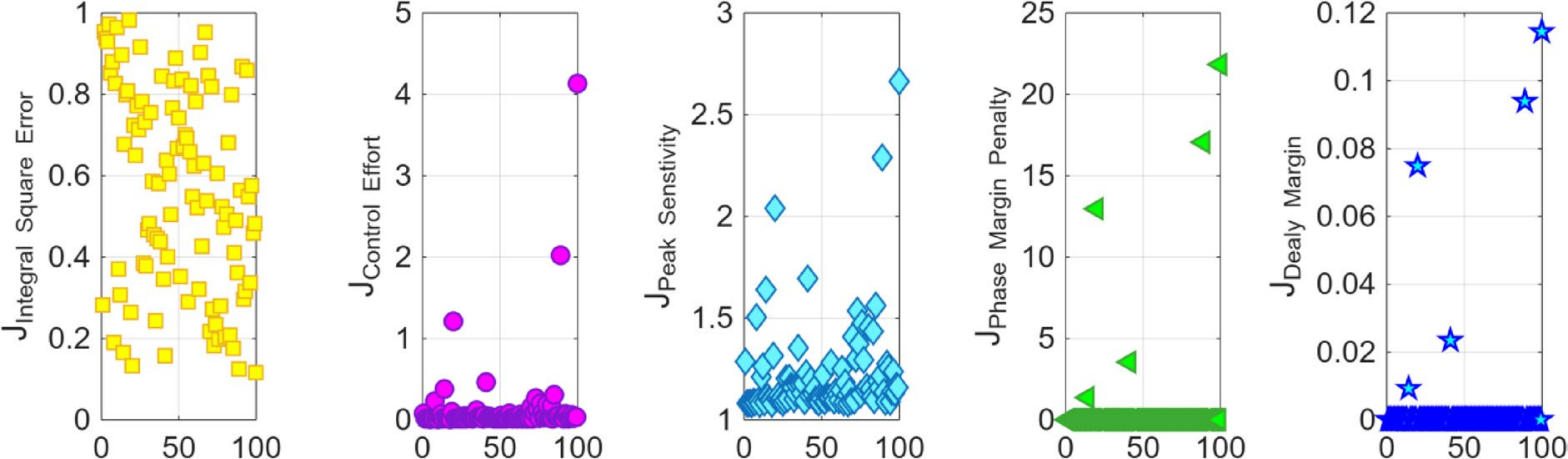
Objective function costs in POS for integrating plant

**Fig. 6:**
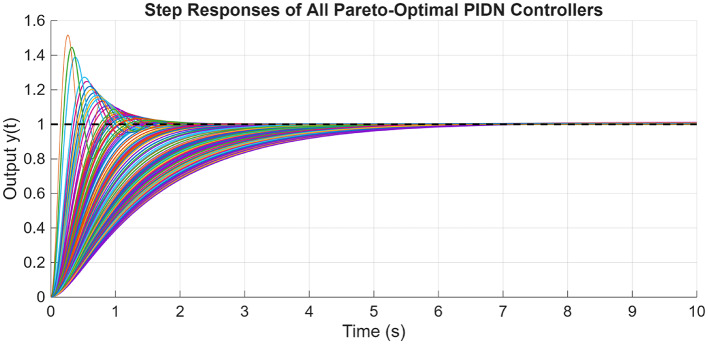
Closed-loop step response for the integrating plant with all controller parameters in POS

**Fig. 7:**
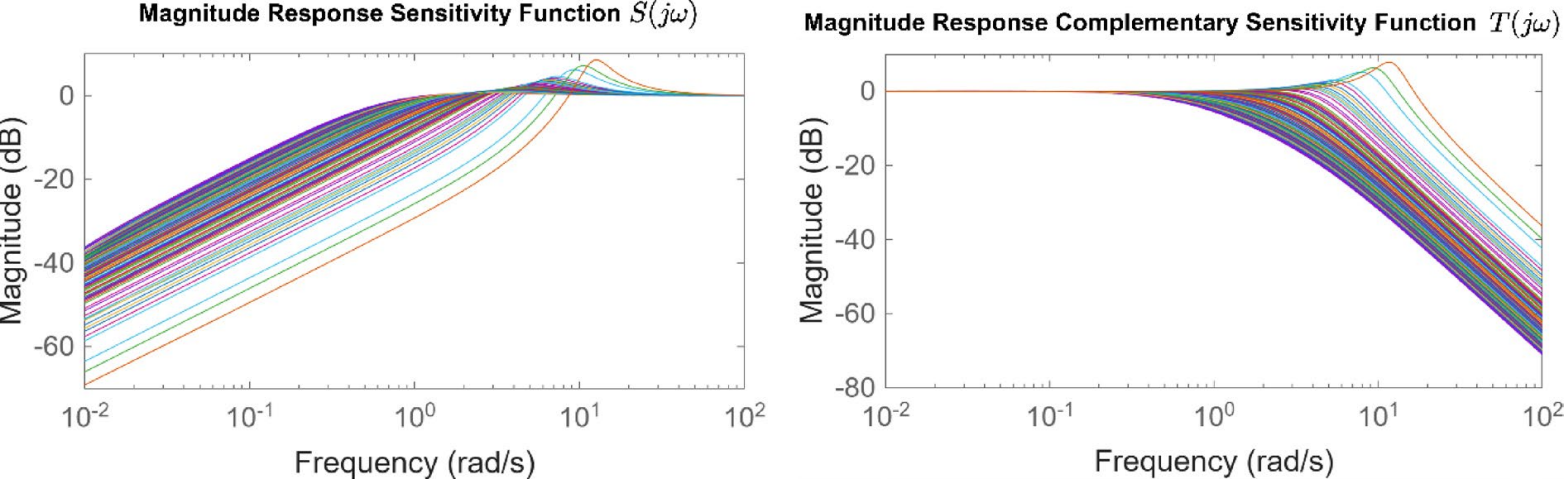
Bode plot for $\:S\left(j\omega\:\right)$ and $\:T\left(j\omega\:\right)$ for integrating plant with all controller parameters in POS

After the optimization process, the POS solutions are obtained, and controller parameters as well as objective function costs obtained in POS are visualized in Figs. 4 and 5 respectively. Figures 6 and 7 shows step and Bode plot of closed loop compensated system respectively; and it is observed that controller offer a stable response.

### Plant 2: DC motor position control

**Fig. 8:**
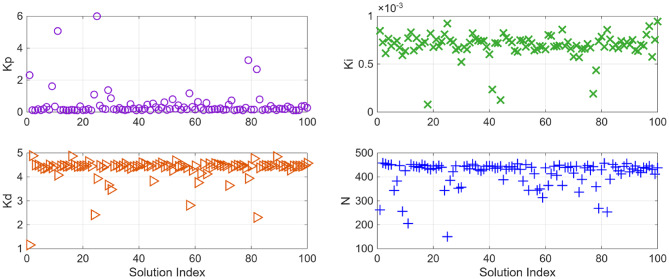
Controller parameters in POS for DC motor position control

**Fig. 9:**
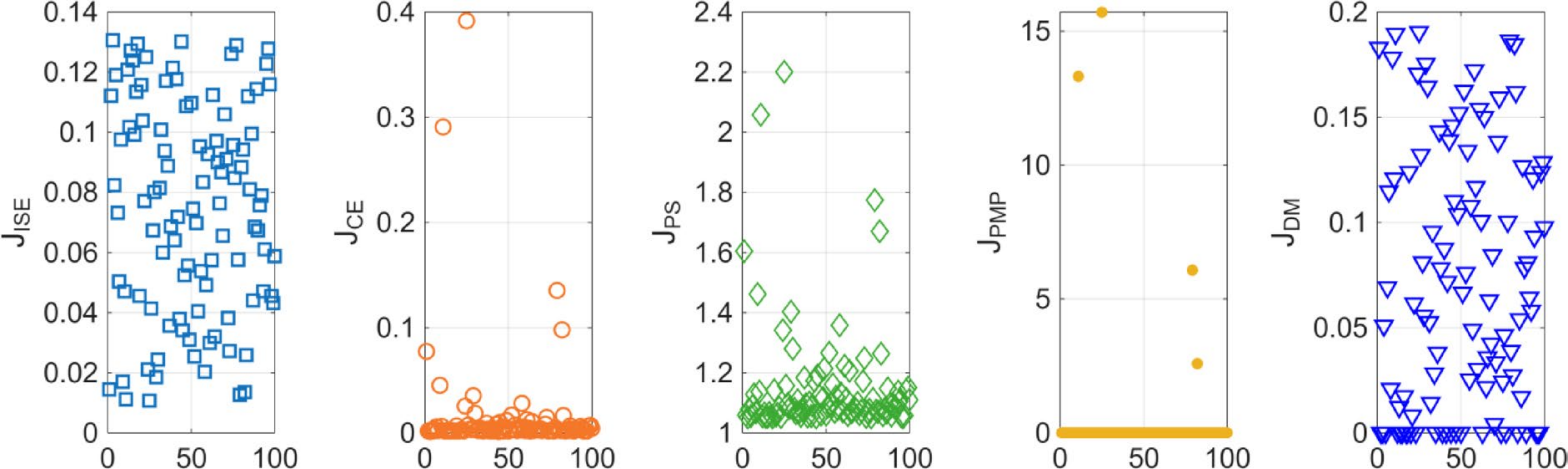
Objective function costs in POS for DC motor position control

**Fig. 10:**
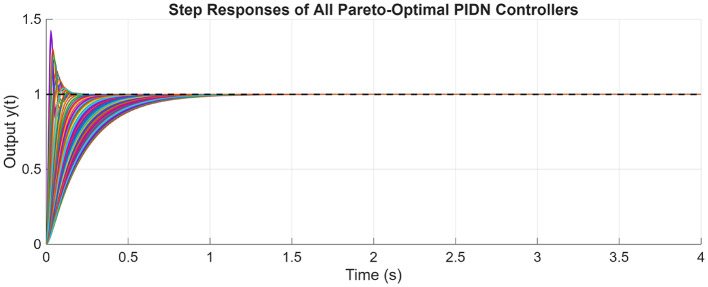
Closed-loop step response for DC motor position control with all controller parameters in POS

**Fig. 11:**
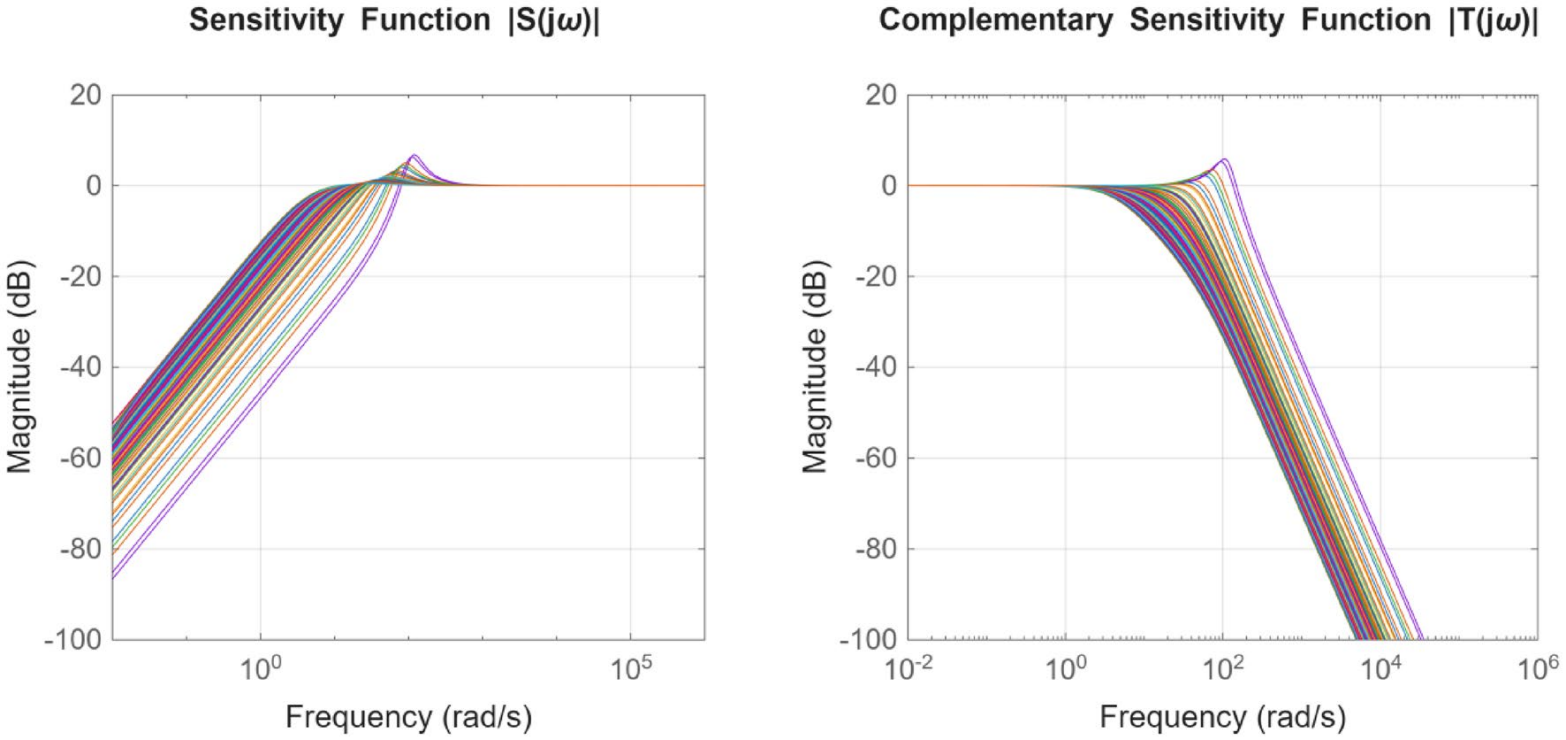
Bode plot for $\:S\left(j\omega\:\right)$ and $\:T\left(j\omega\:\right)$ for DC motor position control with all controller parameters in POS

After the optimization process, the obtained POS solutions and the objective values are visualized in Figs. 8 and 9 respectively. Figures 10 and 11 shows step and Bode plot of closed loop compensated system respectively; and a stable response is attained.

### Plant 3: Non-minimum phase hydropower system

**Fig. 12:**
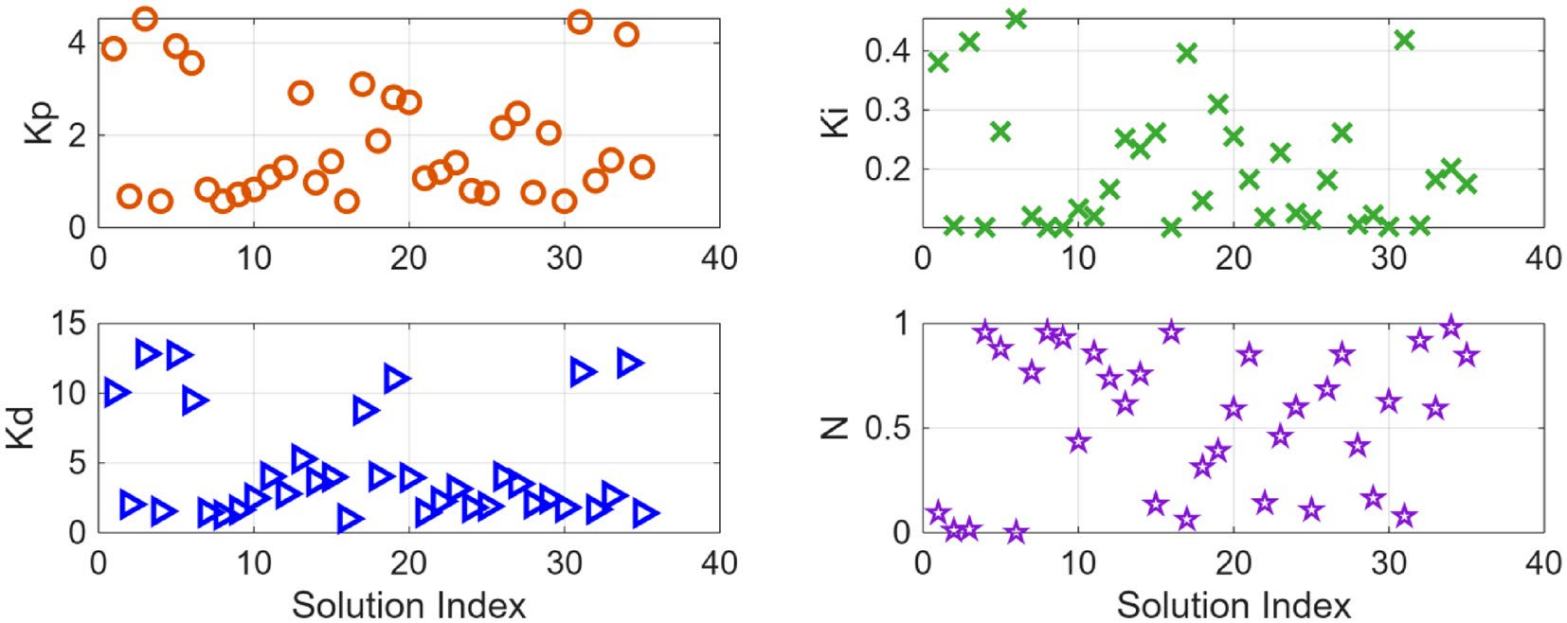
Controller gains in POS for NMP hydropower system

**Fig. 13:**
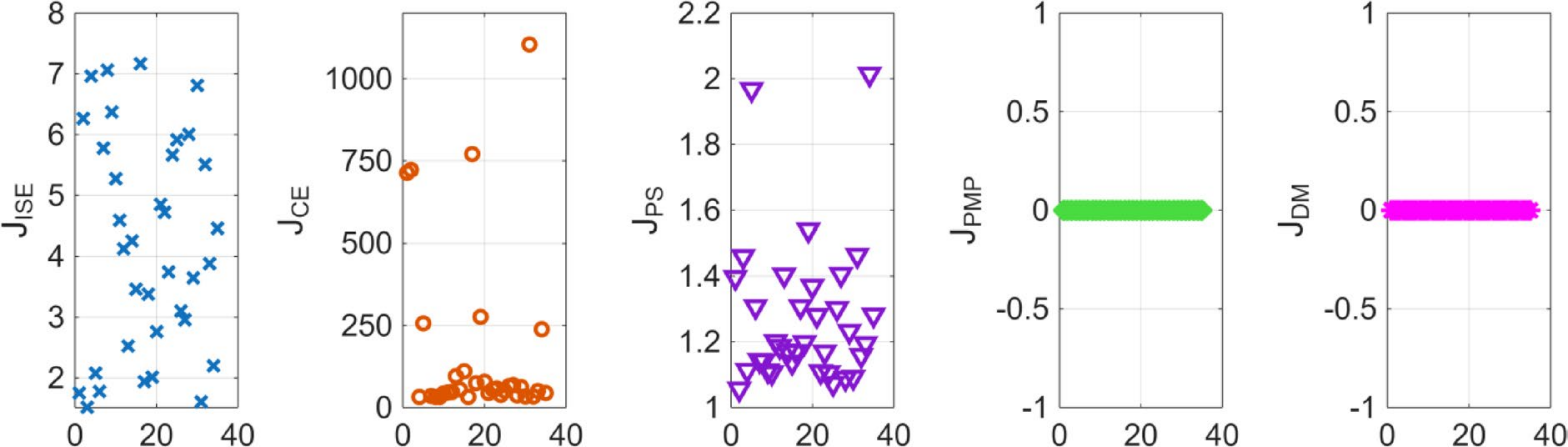
Objective function costs in POS for NMP hydropower system

**Fig. 14:**
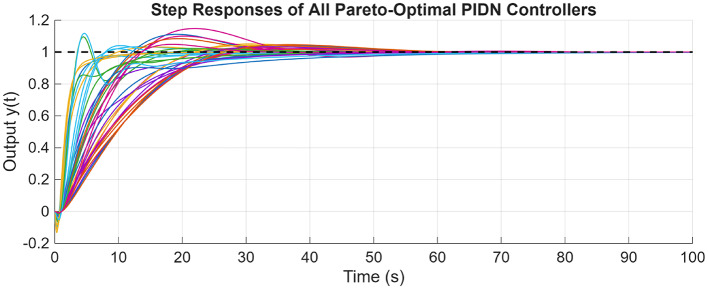
Closed-loop step response for NMP system with all controller parameters in POS

**Fig. 15:**
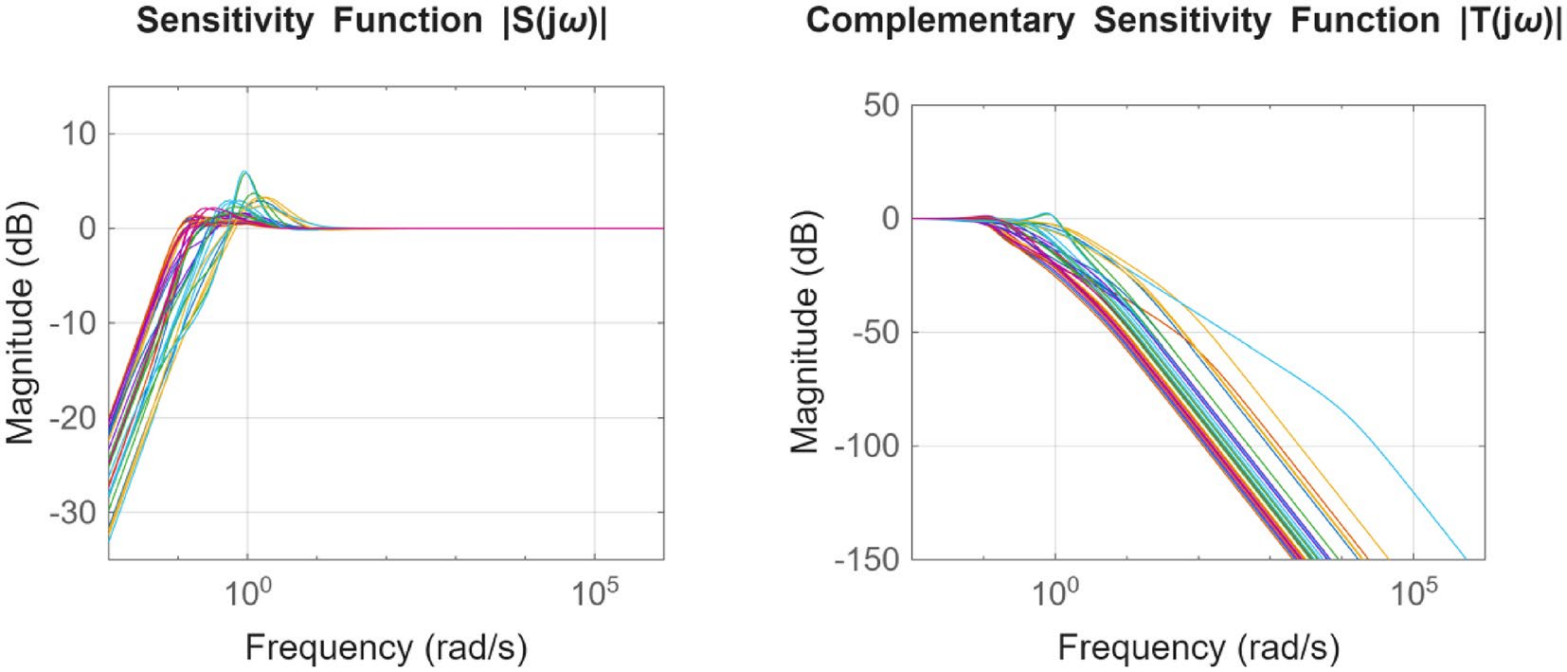
Bode plot for $\:S\left(j\omega\:\right)$ and $\:T\left(j\omega\:\right)$ for NMP system with all controller parameters in POS

Similarly for the non-minimum phase hydropower system, the plot for the POS solutions and the objective values are visualized in Figs. 12 and 13 respectively. Figures 14 and 15 shows the step and Bode plot of the closed loop compensated system respectively; and it can be observed that the controller gains offer a stable response.

### Plant 4: Coupled tank system

**Fig. 16:**
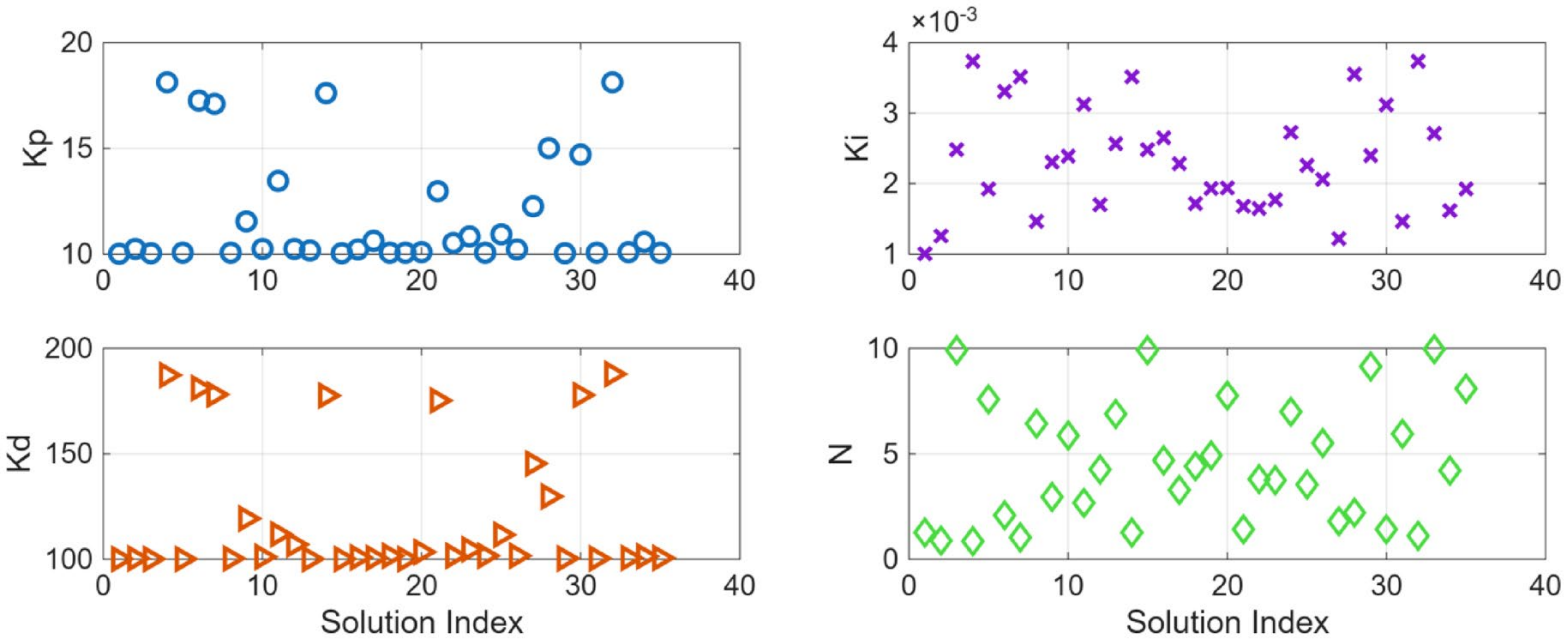
Controller gains in POS for coupled tank system

**Fig. 17:**
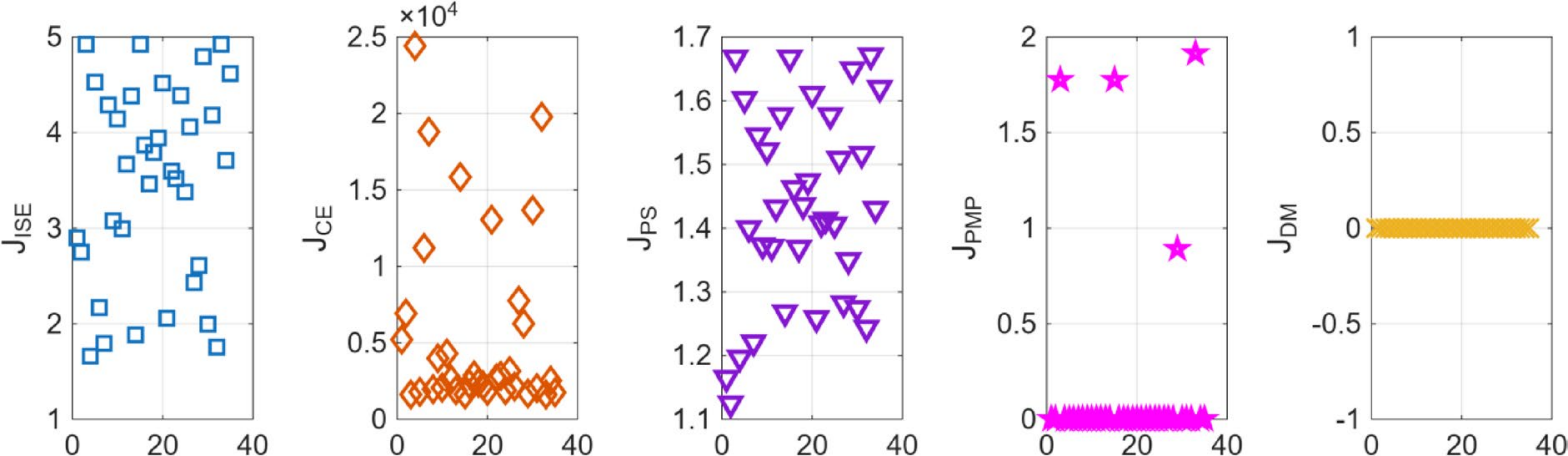
Objective function costs in POS for coupled tank system

**Fig. 18:**
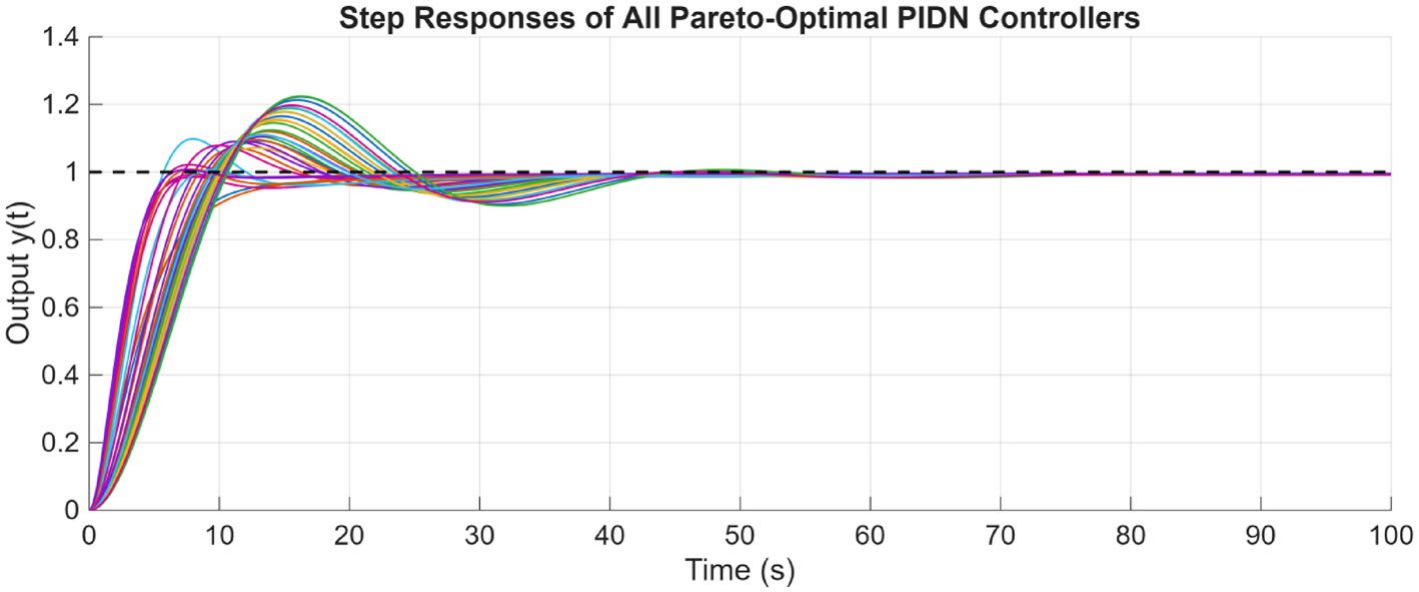
Closed-loop step response for coupled tank system with all controller parameters in POS

**Fig. 19:**
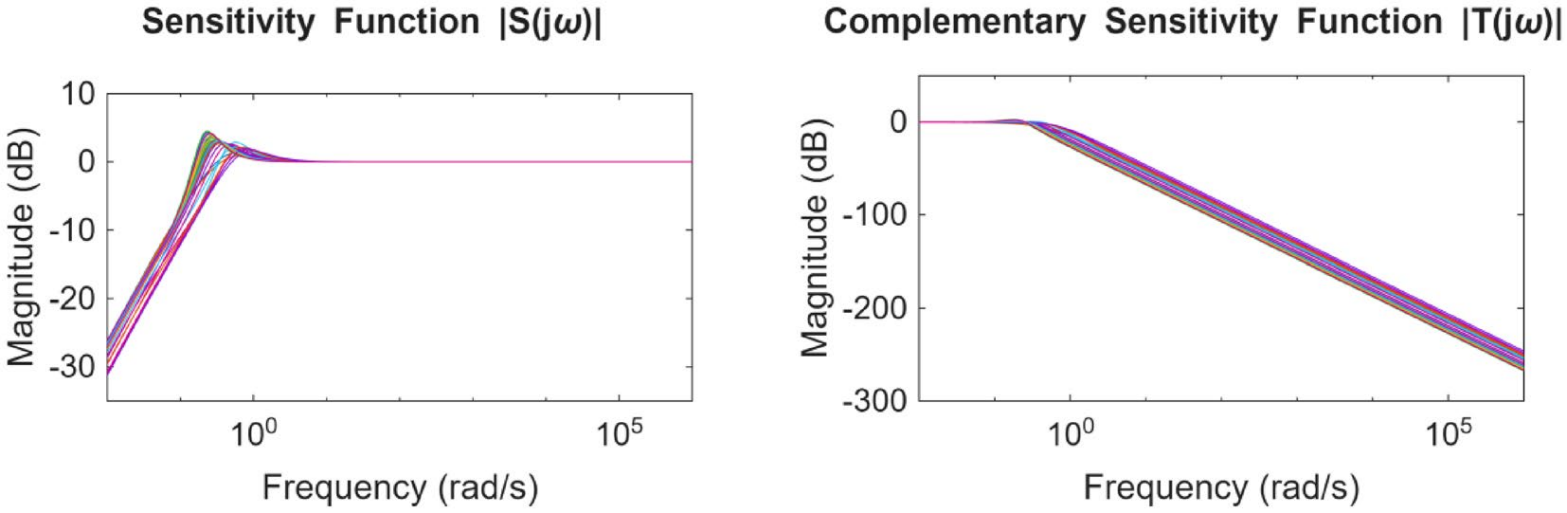
Bode plot for $\:S\left(j\omega\:\right)$ and $\:T\left(j\omega\:\right)$ for coupled tank system with all controller parameters in POS

For the coupled tank system, the POS of decision variables and the objective values is visualized in Figs. 16 and 17. Figures 18 and 19 shows the step and Bode plot of the closed loop compensated system; and it can be observed that the controller gains offer a stable response.

## K-means clustering-based ideal solution selection

The goal of multi-objective optimization is to achieve a simultaneous trade-off among multiple, often conflicting, objectives $\:J\left(x\right)$, and returns a Pareto-optimal set of solutions $\:{x}_{i}$ is given by Eqs. (13) & (14) respectively. Pareto fronts are often used to visualize the POS and choose the trade-off; and offer a very constrained visualization in cases when objectives are greater than three.

13 $$\:\text{m}\text{i}\text{n}\:J\left(x\right)=\left\{{j}_{1}\left(x\right),\:{j}_{2}\left(x\right),...,\:{j}_{m}\left(x\right)\right\},\:\:where\:\:\:\:x\in\:\varOmega\:$$

14 $$\begin{gathered} \:x_{i} = {\mathcal{P}} \Leftrightarrow \:{\nexists }\:x_{j} \in \:\Omega \::\:\:\:\:\:\:f_{k} \left( {x_{j} } \right) \le \:f_{k} \left( {x_{i} } \right)\forall \:k\:\:\:\:\:{\text{and}}\:\:\:\:\:\: \hfill \\ {\text{f}}_{{\text{l}}} \left( {{\text{x}}_{{\text{j}}} } \right) < {\text{f}}_{{\text{l}}} \left( {{\text{x}}_{{\text{i}}} } \right)\:\:\:{\text{for}}\:{\text{at - least}}\:{\text{one}}\:{\text{l}} \hfill \\ \end{gathered}$$

Where, $\:m$ corresponds to the number of objectives, and $\:{\Omega\:}$ is the feasible decision space.

Pareto fronts typically comprise numerous solutions, with many exhibiting close similarity within the objective space. To choose ideal solution from POS, use of distance-to-ideal could introduce bias to the selection of the solutions in the dense regions in-spite of highlighting the distinct trade-off. So, in the proposed work, we have explored the use of K-Means clustering to find the ideal solution from the POS. Clustering is an unsupervised learning technique that groups the similar solutions together based upon the space proximity and redundancy, allowing for a final selection to be diverse and balanced.

Let $\:\mathcal{P}$ is the Pareto optimal set of solutions and is given by Eq. (15):

15 $$\:\mathcal{P}=\left\{{x}_{1},{x}_{2},\dots\:,{x}_{n}\right\},\:\:\:\:{x}_{i}\in\:{\mathbb{R}}^{m}$$

As the objectives can be in different scales, (like, control effort, ISE, peak sensitivity, etc.), it is required to normalize them first in range of [0, 1] to prevent any single objective (large scale) to dominate the clustering process and introduce bias in the solutions. Mathematically given by Eq. (16) as,

16 $$\:{x}_{i,j}^{norm}=\frac{{x}_{i,j}-\text{min}\left({x}_{j}\right)}{\text{max}\left({x}_{j}\right)-\text{m}\text{i}\text{n}\left({x}_{j}\right)}$$

Where, $\:{x}_{i,j}$ is the $\:{j}^{th}$ objective of the $\:{i}^{th}$ solution.

After the normalization, the clustering is performed. In the present work the use of K-Means clustering is considered, wherein the solutions are grouped into K clusters by minimizing the variance within the cluster; and is given mathematically by Eq. (17) as,

17 $$\:\mathop {{\text{min}}}\limits_{{C_{1} , \ldots \:,\:C_{K} }} \sum {\:_{{k = 1}}^{K} } \sum {\:_{{x_{i} \in \:C_{k} }} } \parallel x_{i} - \mu \:_{k} \parallel ^{2}$$

Where, $\:{\mu\:}_{k}$ is the centroid of cluster $\:{C}_{k}$.

In the next stage, the estimation of the distance to the ideal (utopia point) point is done. Utopia point represents the best possible values for each objective, even if no single solution attains all of them simultaneously. For the minimization problem, it is represented by Eq. (18) as,

18 $$\:{z}^{*}=\left({z}_{1}^{*},{z}_{2}^{*},\dots\:,\:{z}_{m}^{*}\right),\:\:\:\:\:\:\:{z}_{j}^{*}=\underset{i}{\text{min}}{f}_{j}\left({x}_{i}\right)$$

The distance-to-ideal offers a quantification of how close a solution is to utopia point; and is estimated using the normalized Euclidean distance, given mathematically by Eq. (19) as:

19 $$\:d\left({x}_{i},{z}^{*}\right)=\sqrt{\sum\:_{j=1}^{m}{\left(\frac{{f}_{j}\left({x}_{i}\right)-{z}_{j}^{*}}{\underset{i}{\text{max}}{f}_{j}\left({x}_{i}\right)-\underset{i}{\text{min}}{f}_{j}\left({x}_{i}\right)}\right)}^{2}}$$

The best solutions from the POS can be selected based on the minimum distance; the solutions that exhibits a minimum distance are closer to the ideal point and offers the best compromise amongst all the objectives; and the best compromise solution that represents the most balanced trade-off is referred as the knee point on the Pareto front; given mathematically by Eq. (20) as:

20 $$\:{x}^{*}=\text{arg}\underset{{x}_{i}\in\:\mathcal{P}}{\text{min}}d\left({x}_{i},{z}^{*}\right)$$

### Integrating plant

**Fig. 20:**
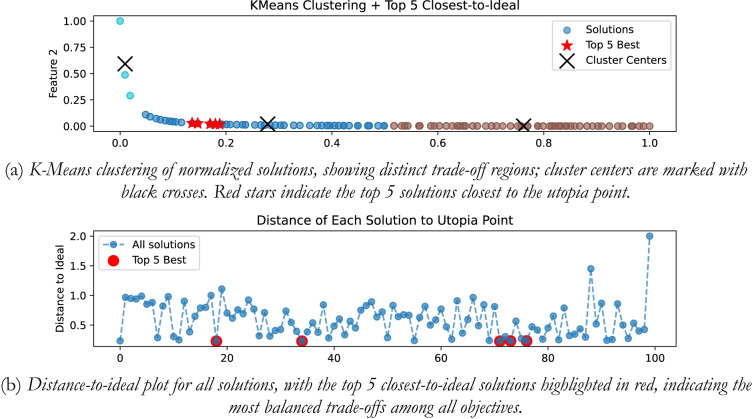
Selection of best-compromise solution from Pareto-optimal set for the Integrating Plant.

**Fig. 21:**
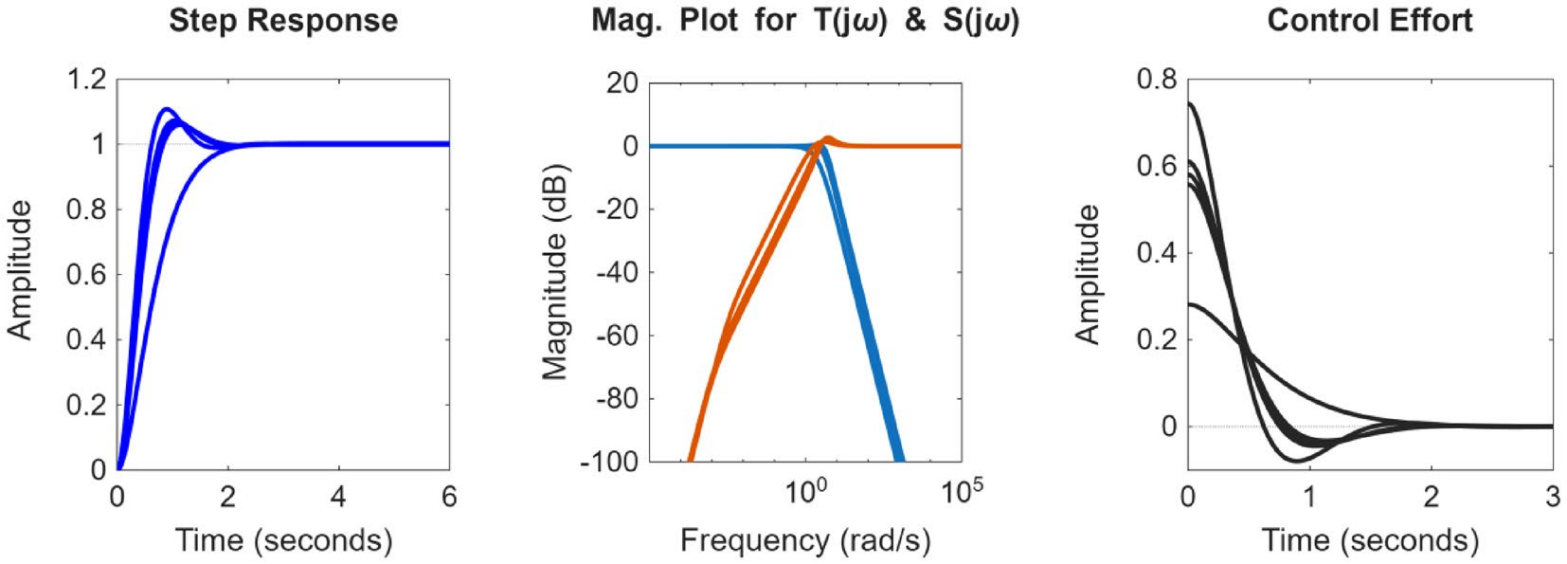
Response of best-compromise solution from POS for the Integrating Plant

In the presented work for all the four plants, K-means clustering is applied to cluster the Pareto optimal set into 3 clusters using k-means + + initialization, Euclidian distance is used, and 300 iterations are considered. Figure 20(a) shows the plot for distinct trade-off regions after applying K means clustering of normalized solutions; black crosses represent the cluster centres and the red stars indicate the top 5 solutions closest to the utopia point; and Fig. 20 (b) shows the plot for the distance to the ideal for all the solutions; and the top 5 closet-to-the-ideal solutions are highlighted in red color, offering the most balanced trade-off amongst objectives. Table 1 shows the best possible controller gains as well as the closed loop time and frequency domain metrics for best-compromise solution from POS for integrating plant. Figure 21 shows plot for step response, magnitude plot for sensitivity and complementary sensitivity functions and control efforts for the top 5 solutions in the POS.

**Table 1:** Controller gains and performance indices for best-compromise solution from POS for integrating Plant.

	$\:{\varvec{x}}_{1}$	$\:{\varvec{x}}_{2}$	$\:{\varvec{x}}_{3}$	$\:{\varvec{x}}_{4}$	$\:{\varvec{x}}_{5}$
Proportional Gain$\:{K}_{P}$	0.586	0.274	0.566	0.727	0.54
Integral Gain$\:{K}_{I}$	0.000691	0.00091	0.00071	0.000784	0.000746
Derivative Gain$\:{K}_{D}$	6.95	2.18	4.24	4.38	4.1
Derivative Filter$\:N$	289	282	397	259	244
Rise Time (sec.)	0.5005	1.1554	0.5251	0.4188	0.5458
Settling Time (sec.)	1.5395	1.9203	1.5826	1.3702	1.6161
Overshoot %age	7.3386	0.2306	6.5394	10.7237	5.9197
Steady State Error	0	0	0	0	0
Peak Sensitivity	1.3187	1.1707	1.3062	1.3723	1.2965
Peak Complementary Sensitivity	1.0168	1.0012	1.0004	1.0058	1.0004

### Position control of DC motor

**Fig. 22:**
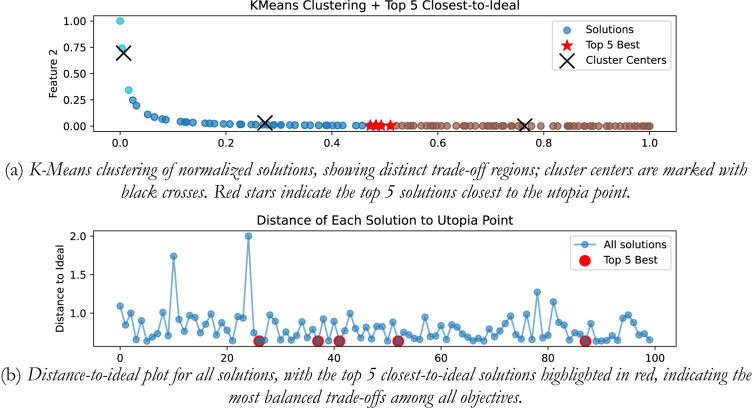
Selection of best-compromise solution from POS for position control of DC motor.

**Fig. 23:**
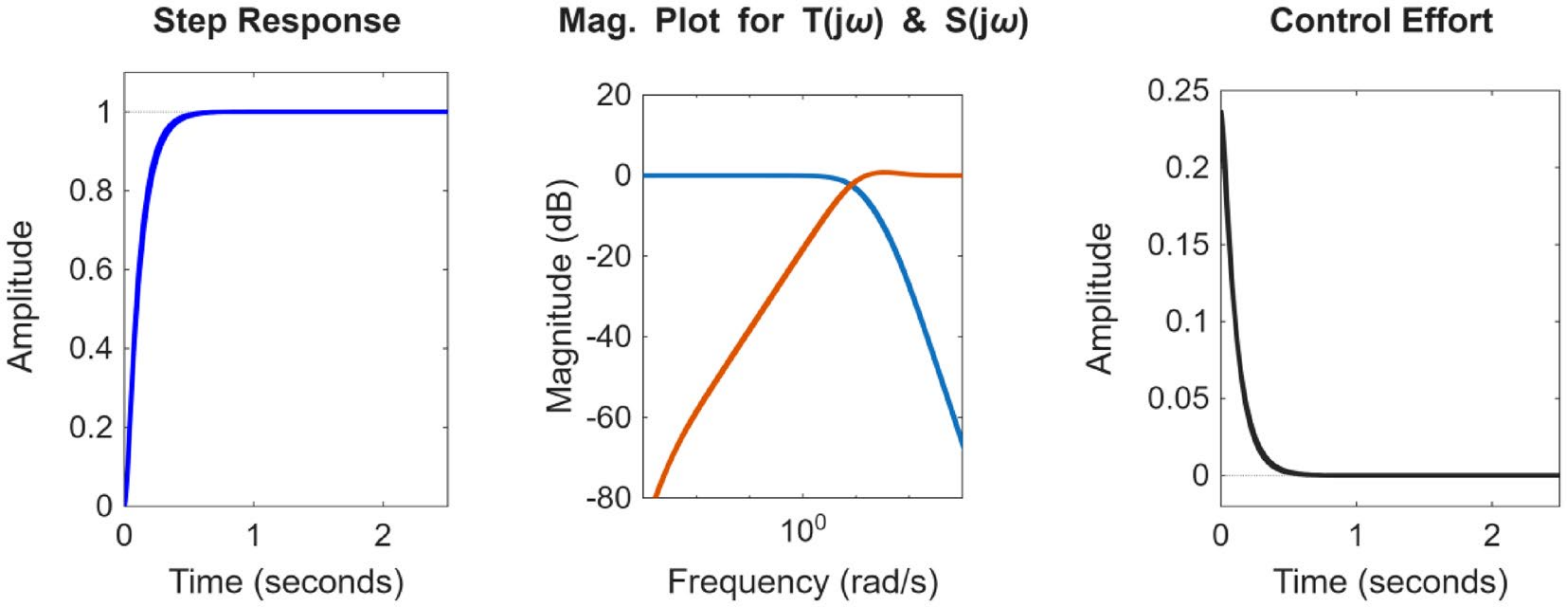
Response of best-compromise solution from POS for position control of the DC motor

The same algorithm specific parameters are used for performing the clustering as discussed above. Figure 22 (a) and 22 (b) shows the plot for distinct trade-off regions after applying K means clustering of normalized solutions and the plot for the distance to the ideal for all the solutions. Table 2 shows the best possible controller gains as well as the closed loop performance metrics. Figure 23 shows the plot for the step response, the magnitude plot for sensitivity and complementary sensitivity functions and the control efforts for the top 5 solutions in the POS.

**Table 2:** Controller gains and performance metrics for the best-compromise solution from POS for the position control of the DC Motor.

	$\:{\varvec{x}}_{1}$	$\:{\varvec{x}}_{2}$	$\:{\varvec{x}}_{3}$	$\:{\varvec{x}}_{4}$	$\:{\varvec{x}}_{5}$
Proportional Gain $\:{K}_{P}$	0.217	0.221	0.209	0.222	0.226
Integral Gain $\:{K}_{I}$	0.000681	0.000743	0.00072	0.000693	0.000729
Derivative Gain $\:{K}_{D}$	4.67	4.42	4.55	4.47	4.39
Derivative Filter $\:N$	451	432	436	443	422
Rise Time (sec.)	0.2327	0.2279	0.2419	0.2273	0.222
Settling Time (sec.)	0.4222	0.4135	0.4388	0.4124	0.4028
Overshoot %age	0.0232	0	0	0	0.0083
Steady State Error	0	0	0	0	0
Peak Sensitivity	1.0935	1.095	1.0909	1.0952	1.0968
Peak Complementary Sensitivity	1.0002	1.0002	1.0002	1.0002	1.0002

### Hydro power system

**Fig. 24:**
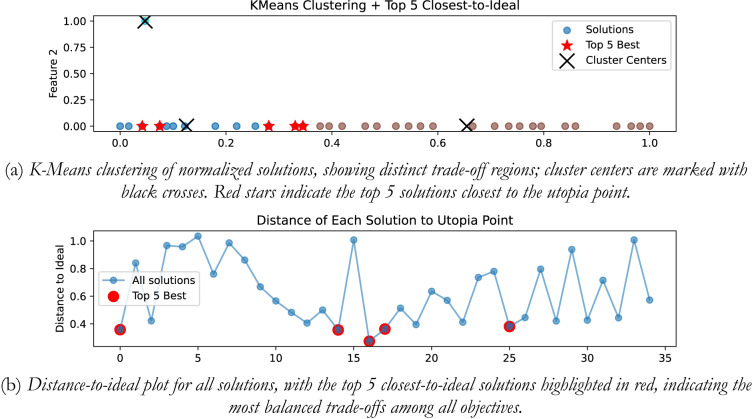
Selection of best-compromise solution from Pareto-optimal set for the Hydro Power System.

**Fig. 25:**
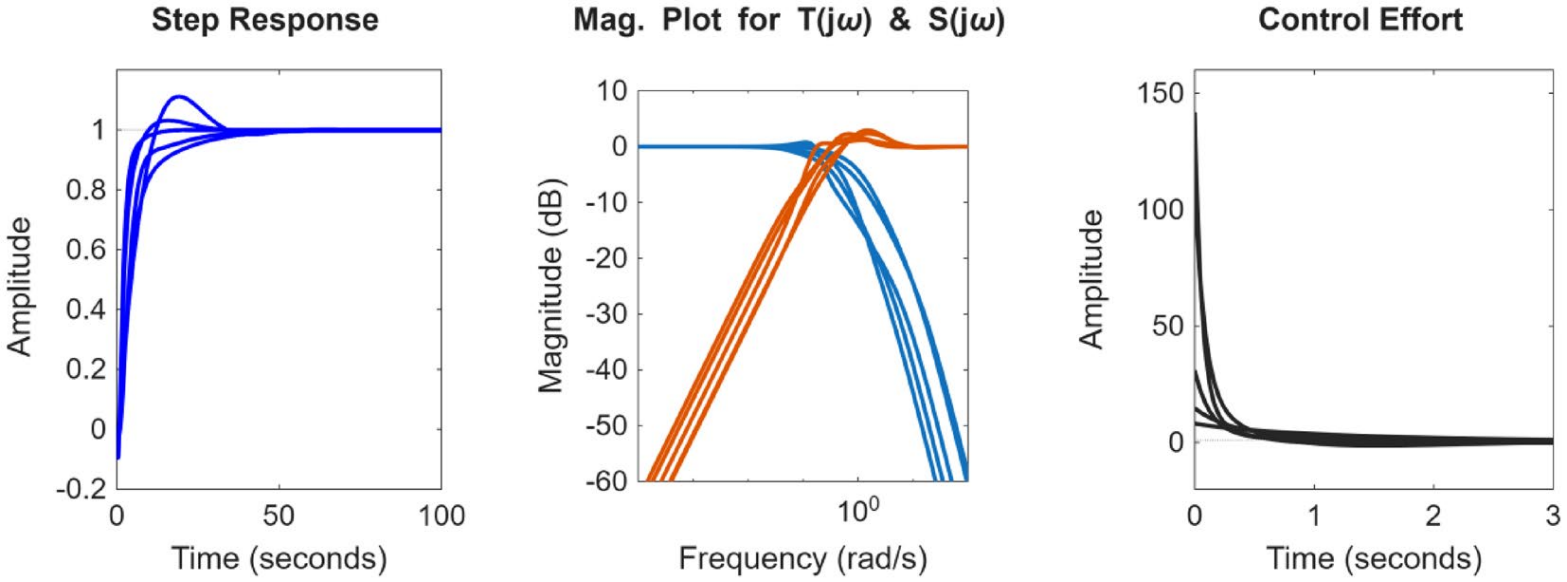
Response of best-compromise solution from POS for the Hydro Power System

Figure 24 (a) and 24 (b) shows the plot for distinct trade-off regions after applying K means clustering of normalized solutions and the plot for the distance to the ideal for all the solutions for the non-minimum phase hydropower system. Table 3 shows the best possible controller gains as well as the closed performance metrics. Figure 25 shows the plot for the step response, the magnitude plot for sensitivity and complementary sensitivity functions and the control efforts for the top 5 solutions in the POS.

**Table 3:** Controller gains and performance indices for best-compromise solution from POS for the hydro power System

	$\:{\varvec{x}}_{1}$	$\:{\varvec{x}}_{2}$	$\:{\varvec{x}}_{3}$	$\:{\varvec{x}}_{4}$	$\:{\varvec{x}}_{5}$
Proportional Gain $\:{K}_{P}$	3.11	1.44	3.88	1.87	2.17
Integral Gain $\:{K}_{I}$	0.396	0.261	0.38	0.146	0.182
Derivative Gain $\:{K}_{D}$	8.78	3.99	10	4.06	4.06
Derivative Filter $\:N$	0.0633	0.135	0.0935	0.315	0.686
Rise Time (sec.)	4.8554	8.3965	3.5937	12.9846	7.2611
Settling Time (sec.)	22.1563	30.9705	9.9476	36.1225	28.6976
Overshoot %age	3.1812	11.1360	0	0	0
Undershoot %age	8.4939	3.4272	9.5255	2.9121	2.2648
Steady State Error	0	0	0	0	0
Peak Sensitivity	1.3096	1.1382	1.3973	1.1979	1.3031
Peak Complementary Sensitivity	1.0181	1.0962	1.00	1.00	1.00

### Coupled tank system

**Fig. 26:**
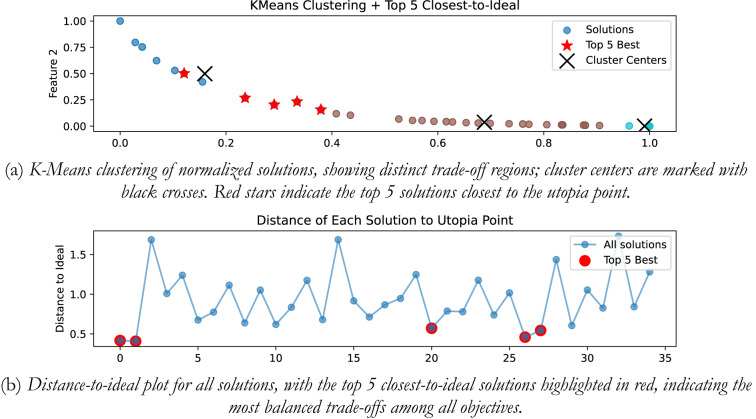
Selection of best-compromise solution from Pareto-optimal set for coupled tank systems

**Fig. 27:**
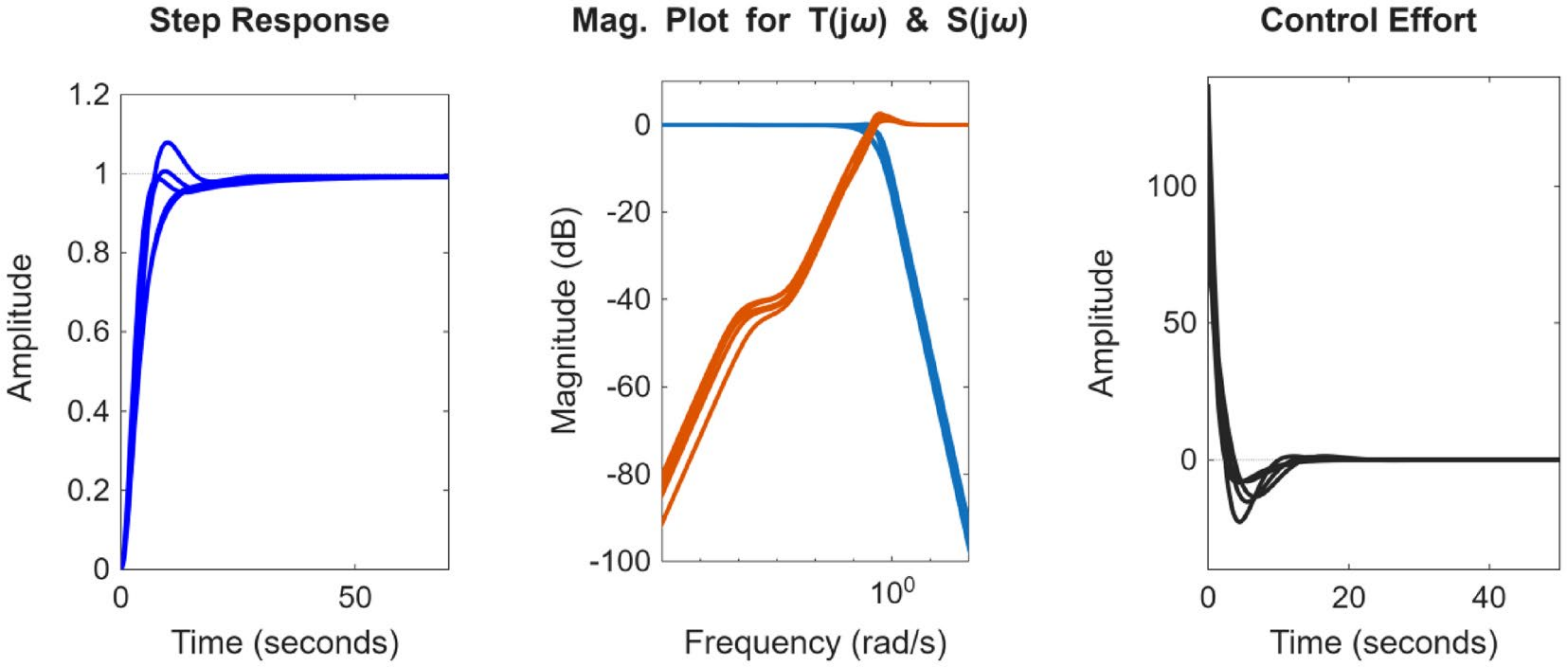
Response of best-compromise solution from POS for coupled tank systems.

Figure 26 (a) and 26 (b) shows the plot for distinct trade-off regions after K means clustering and the plot for the distance to the ideal for all the solutions for coupled tank system. Table 4 shows best possible controller gains as well as the closed loop performance metrics. Figure 27 shows the plot for the step response, the magnitude plot for sensitivity and complementary sensitivity functions and the control efforts for the top 5 solutions in the POS.

**Table 4:** Controller gains and performance indices for the best-compromise solution from POS for coupled tank systems.

	$\:{\varvec{x}}_{1}$	$\:{\varvec{x}}_{2}$	$\:{\varvec{x}}_{3}$	$\:{\varvec{x}}_{4}$	$\:{\varvec{x}}_{5}$
Proportional Gain $\:{K}_{P}$	10.2	10	12.3	15	13
Integral Gain $\:{K}_{I}$	0.00126	0.001	0.00122	0.00355	0.00168
Derivative Gain $\:{K}_{D}$	100	100	145	130	175
Derivative Filter $\:N$	0.895	1.26	1.79	2.2	1.41
Rise Time (sec.)	8.64	7.9159	4.9727	4.8256	4.3516
Settling Time (sec.)	20.9581	24.6658	28.8044	20.2477	31.7337
Overshoot %age	0	0	0.6186	7.8416	0
Steady State Error	0	0	0	0	0
Peak Sensitivity	1.00	1.00	1.00	1.0217	1.00
Peak Complementary Sensitivity	1.1246	1.1659	1.2826	1.3495	1.2626

## Comparison with existing methods

The obtained controllers in Sect. 5 are compared with the existing control methods; where PID controller is tuned using three different tuning methods. First, PID controllers are synthesised with Ziegler Nichols method. Secondly, optimal synthesis of the controller is done considering the error-based objective of the minimization of ISE using genetic algorithm (GA), to ensure good time domain tracking behaviour. In the obtained results, it is observed that the ZN and GA-ISE optimized controller didn’t offer good frequency domain behaviour; the ZN controllers offered an oscillatory response; whereas the GA-ISE tuned controller showcase poor frequency domain characteristics.

### Integrating plant

The controller gains obtained using ZN, GA-ISE and best gains in POS are given in Table 5. The work is also compared with the robust controllers proposed in^26^; and the controller expressions are given by Eqs. (21) and (22) as under as:

21 $$\begin{gathered} \:K_{{QFT - M}} = 9.01\frac{{\left( {\frac{s}{{113.8}} + 1} \right)\left( {\frac{s}{{1.1}} + 1} \right)}}{{\left( {\frac{s}{{42.81}} + 1} \right)\left( {\frac{{s^{2} }}{{10^{6} }} + \frac{{1486s}}{{10^{6} }} + 1} \right)}}\: \hfill \\ \:F_{{QFT - M}} = \frac{1}{{\left( {\left( {\frac{s}{{3.752}}} \right)^{2} + \frac{{1.326s}}{{2.752}} + 1} \right)}} \hfill \\ \end{gathered}$$

22 $$\begin{gathered} K_{{QFT - PSV}} = 10.95\frac{{\left( {\frac{s}{{2.1}} + 1} \right)}}{{\left( {\frac{s}{{950}} + 1} \right)\left( {\frac{s}{{982}} + 1} \right)}}\:\: \hfill \\ F_{{QFT - PSV}} = \frac{1}{{\left( {\frac{s}{{3.197}} + 1} \right)\left( {\frac{s}{{8.61}} + 1} \right)}} \hfill \\ \hfill \\ \end{gathered}$$

**Fig. 28:**
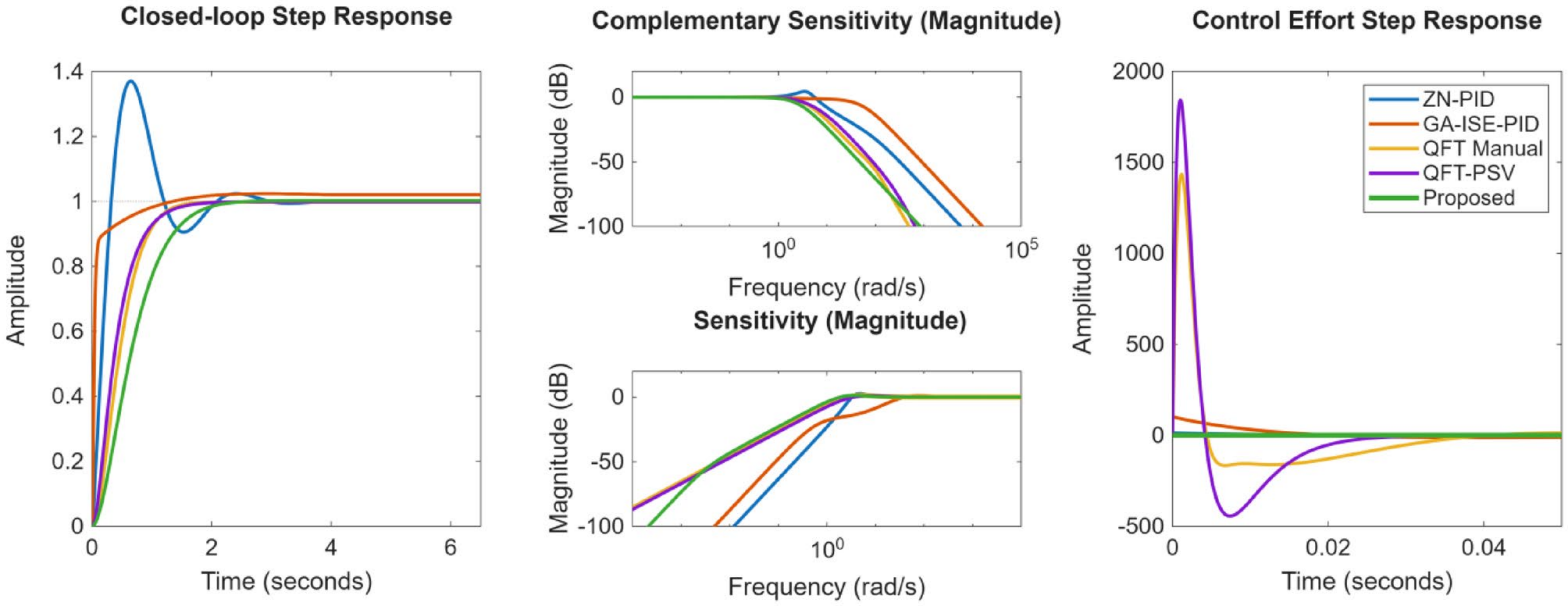
Comparison of the response for integrating plant.

The compared closed loop response and control effort is presented in Fig. 28; and the performance metrics are presented in Table 6. From Fig. 26; Table 6, it is evident that proposed controllers offer better performance characterization as well as exhibit a minimum control effort. While for ZN-PID controllers, very high overshoot of ~ 37% is observed. Also, for of GA-ISE-PID controllers, control effort is too high. In case of robust controllers proposed in^26^, they offer good time and frequency domain characteristics but the required control effort is too large; also characterise high order controllers. The proposed controller offers improved performance characteristics as well as exhibit a minimum control effort.

**Table 5:** Comparison of the controller gain values for integrating plant.

	Kp	Ki	Kd	*N*
ZN Method	1.2	3	0.12	-
GA – ISE	1.4943	0.4810	1.0000	-
Proposed	0.274	0.00091	2.18	282

**Table 6:** Comparison of the performance evaluation metrics for integrating plant.

	ZN Method	GA – ISE	QFT Example	PSV Nataraj [26]	Proposed
Rise Time	0.2453	0.1879	0.797	0.7835	1.1589
Settling Time	2.5817	1.0238	1.3504	1.4328	1.9275
Overshoot %age	37.0081	2.3805	0	0	0.2288
SSE	0	0	0	0	0
Peak Complementary Sensitivity	1.6754	1.0225	1	1	1.0012
Peak Sensitivity	1.3873	1.1399	1.188	1.138	1.1707

### Position control of DC motor

Likewise, operation of proposed controller is compared with ZN, GA-ISE tuned PID controllers and classical $\:{H}_{\infty\:}$ controller ($\:{K}_{H\infty\:}$) (Eq. (23)) and the controller gains are given in Table 7.

23 $$\:{K}_{H\infty\:}=\frac{28.73{s}^{4}+4.18\times\:{10}^{7}{s}^{3}+4.425\times\:{10}^{10}{s}^{2}+2.2475\times\:{10}^{12}s+3.386\times\:{10}^{8}}{{s}^{5}+1.455\times\:{10}^{6}{s}^{4}+1.466\times\:{10}^{9}{s}^{3}+2.104\times\:{10}^{11}{s}^{2}+1.314\times\:{10}^{13}s+6.57\times\:{10}^{10}}$$

**Table 7:** Comparison of the controller gain values.

	Kp	Ki	Kd	*N*
ZN Method	1.983	59.01	0.01675	
GA – ISE	4.8092	0.0195	0.0504	-
Proposed	0.221	0.000743	4.42	432

**Fig. 29:**
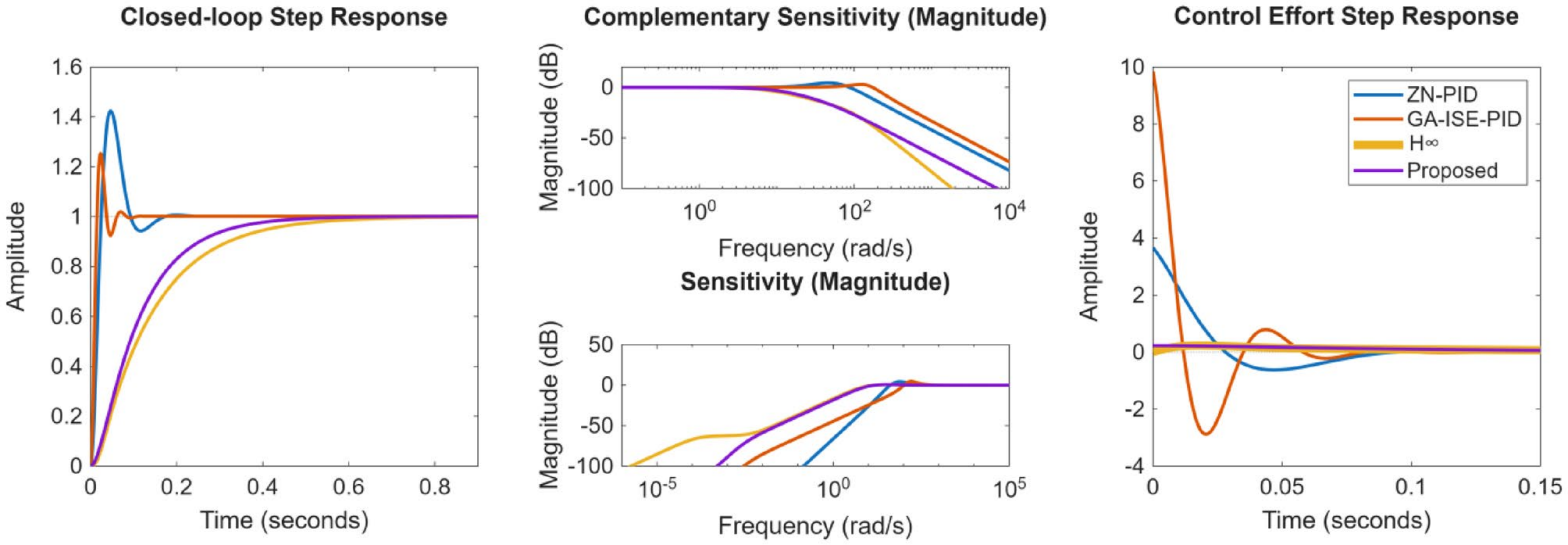
Compared response for position control of DC motor.

The closed loop response as well as the control effort is presented in the Fig. 29; and the various performance evaluation metrics are provided in Table 8. It is evident from the results, that proposed method present better closed loop characteristics as well as minimal control effort; where it is noticed that time domain response of proposed controller is properly damped and does not exhibit any overshoot, where as in case of ZN-PID controller, the overshoots of the magnitude of ~ 42% are evident; whereas the GA-ISE controller offers a very high control effort. Also, when compared to the classical $\:{H}_{\infty\:}$ controller, proposed controller offers better closed loop response characterization.

**Table 8:** Comparison of the performance evaluation metrics for integrating plant.

	ZN Method	GA – ISE	$\:{H}_{\infty\:}$	Proposed
Rise Time	0.017	0.010	0.2907	0.2279
Settling Time	0.1511	0.0576	0.5375	0.4138
Overshoot %age	42.4041	25.3429	0	0
SSE	0	0	0	0
Peak Complementary Sensitivity	1.6563	1.3836	1	1.0002
Peak Sensitivity	1.6021	1.6840	1.093	1.095

### Non-minimum phase hydro power plant

**Fig. 30:**
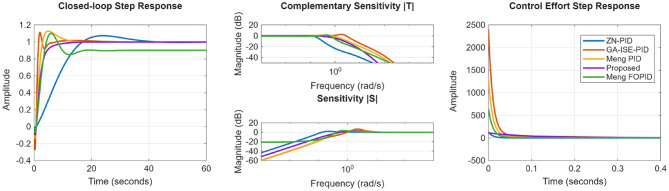
Comparison of the response for non-minimum phase plant.

Next, performance is evaluated for NMP hydro power systems wherein proposed controller is compared with the ZN, GA-ISE tuned PID controllers, the fractional order QFT controllers proposed by Li Meng in^27^. The controller gains are specified in Table 9. The compared closed loop response as well as the control effort is presented in the Fig. 30; and the various performance metrics are given in Table 10. From the obtained results, is observed that in case of GA-ISE-PID controller and controllers proposed in^27^, the overshoots of the magnitude of ~ 7% – 20% are evident along with a very high control effort. Thus, the proposed method offers better closed loop characteristics as well as minimal control effort; where it is noticed that time domain response with proposed controller is properly damped and does not exhibit any overshoot.

**Table 9:** Comparison of the controller gain values for non-minimum phase hydropower plant.

	Kp	Ki	Kd	*N*
ZN Method	0.90984	0.1483	1.2928	-
GA – ISE	7.2140	0.9452	23.9717	-
Fractional Order – PID L. Meng [27]	4.744	1, λ = 0.370	6.493, µ = 1	-
PID L. Meng [27]	6.697	1	12.828	-
Proposed	3.88	0.38	10	0.0935

**Table 10:** Comparison of the performance evaluation metrics for non-minimum phase plant.

	ZN Method	GA – ISE	L. Meng (FOPID) [27]	L. Meng (PID) [27]	Proposed
Rise Time	11.509	0.684	1.635	2.298	3.5892
Settling Time	36.022	5.0029	11.732	16.55	9.8692
Overshoot %age	7.312	11.32	13.782	20.792	0
SSE	0.0035	0.0014	9.73	0	0
Undershoot %age	1.127	27.151	13.782	7.623	9.459
Peak Sensitivity	1.228	2.179	1.522	1.206	1.3957
Peak Complementary Sensitivity	1.0229	1.3599	1.1541	1.5185	1.0

### Coupled tank systems

Finally, the performance is evaluated for the coupled tank systems and controller gains are given in Table 11. The work is also compared with a QFT controller proposed in^25^ and is given by Eq. (24) as:

24 $$\begin{gathered} K_{{QFT}} \left( s \right) = \frac{{2.22 \times \:10^{7} s^{4} + 1.85 \times \:10^{6} s^{3} + 3.43 \times \:10^{4} s^{2} + 82.69s + 0.053}}{{6.94 \times \:10^{4} s^{6} + 2.89 \times \:10^{5} s^{5} + 3.80 \times \:10^{5} s^{4} + 1.63 \times \:10^{5} s^{3} + 2630s^{2} + 2s}} \hfill \\ F_{{QFT}} = \frac{1}{{60s + 1}}\: \hfill \\ \end{gathered}$$

**Fig. 31:**
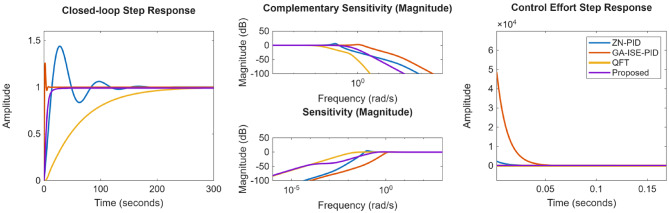
Comparison of the response for coupled tank system.

Compared closed loop response as well as control effort is presented in the Fig. 31; and the various performance metrics are specified in Table 12. From Fig. 31, it is observed that the ZN-PID controller offers an oscillatory response, where high overshoots of the order of ~ 43% are observed. In the case of GA-ISE-PID controllers, a fast response is evident but it has been realized at a cost of higher overshoot of ~ 25% and high control effort. In the case of QFT controllers proposed in^25^, a very high order controller is realized and offers a slower response. Thus, results specify that proposed method bids better performance characteristics as well as minimal control effort; where it can be noticed that time domain characteristics with proposed controller is properly damped and peak values for sensitivity and complementary sensitivity are nearer to unity further ratifies the robustness characteristics.

**Table 11:** Comparison of the controller gain values for coupled tank system.

	Kp	Ki	Kd	*N*
ZN Method	7.207	0.4562	28.471	-
GA – ISE	745.9340	1.0117	599.9999	-
Proposed	10.2	0.00126	100	0.895

**Table 12:** Comparison of the performance evaluation metrics for coupled tank system.

	ZN Method	GA – ISE	QFT [25]	Proposed
Rise Time	10.908	0.786	130.508	8.687
Settling Time	137.383	5.926	236.065	21.091
Overshoot %age	43.913	25.658	0	0
SSE	1.9673	0.0046	0	0
Peak Sensitivity	1.722	1.1148	1.00	1.00
Peak Complementary Sensitivity	1.967	1.3748	1.0511	1.124

## Performance evaluation with parametric uncertainties

To inspect the robustness of proposed controllers; parametric uncertainties have been considered; and are given by Eqs. 25, 26, 27 and 28. The closed loop responses are given in Fig. 33. It is observed from the proposed controllers offers the time responses in a very tight envelope even in presence of parametric uncertainties; establishing their robustness of the proposed controller. In the case of position control of DC motor, it observed that only proposed and $\:{H}_{\infty\:}$ controllers exhibit good response, whereas a lot of variation can be observed for the ZN and GA-ISE tuned controllers and is evident in Fig. 33(b). Also, in case of the NMP hydro-power systems, only proposed controller exhibits a non-oscillatory behavior as can be observed in Fig. 33(c). Finally, for coupled tank system, proposed controller offers a satisfactory response, whereas large variations can be observed in case of ZN and GA-ISE controllers, the QFT controller offer a robust response but is sluggish and can be observed in Fig. 33(d).

25 $$\:{G}_{INT}\left(s\right)=\frac{ka}{s\left(s+a\right)};\:\left\{k,\:a\in\:\pm\:20\%\, \text{of}\,5\right\}$$

$$\:{G}_{DC\:Motor}\left(s\right)=\frac{K}{s\left(\left(Js+b\right)\left(Ls+R\right)+{K}^{2}\right)};$$

26 $$\:\left\{K,J,b,L,R\right\}\in\:\pm\:10\%\:\text{of}\:\left\{0.0274,\:3.2284\times\:{10}^{-6},\:3.5077\times\:{10}^{-6},\:2.75\times\:{10}^{-6},\:4\:\right\}$$

27 $$\:{G}_{NMP-HPS}\left(s\right)=\frac{\left(1-{T}_{w}s\right)}{\left(1+{T}_{g}s\right)\left(1+0.5{T}_{w}s\right)\left(D+2Hs\right)};\left\{D\in\:\pm\:5\%\,\text{of 1},\:H\in\:\pm\:10\%\,\text{of 3}\right\}$$

28 $$\:{G}_{CTank}\left(s\right)=\frac{N}{X{s}^{2}+Ys+1};\:\left\{N,X,Y\in\:\pm\:20\%\,\text{of 10.2, 5000, 510}\right\}$$

**Fig. 32:**
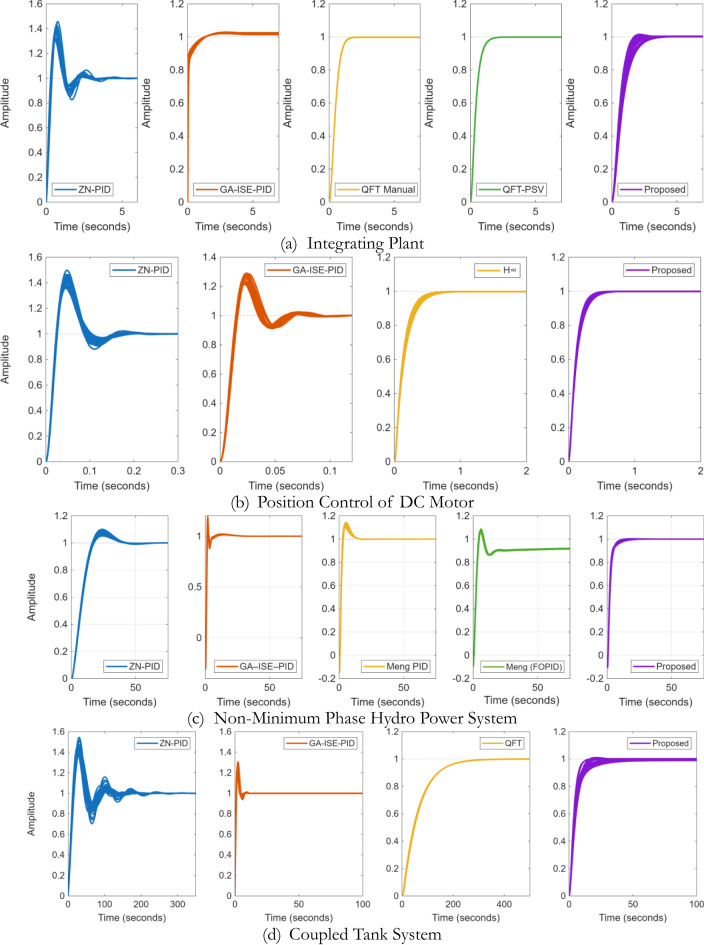
Comparison of the step response for parametrically uncertain plants.

## Robustness analysis via. Monte Carlo simulations

To evaluate the robustness of the proposed controllers against the parametric uncertainties, Monte Carlo simulations have been conducted for all the plants and the performance of the proposed controllers are compared against the existing ones. The plant characterizes uncertain parameters which are uniformly distributed random numbers around their nominal values. An uncertain parameter vector $\:\theta\:$ can be expressed mathematically by Eq. (29).

29 $$\:\varvec{\theta\:}=[{\theta\:}_{1},{\theta\:}_{2},\dots\:,{\theta\:}_{m}{]}^{T},{\theta\:}_{i}={\theta\:}_{i,\text{nom}}(1+{\delta\:}_{i}),$$

Where, the normalized uncertainty $\:{\delta\:}_{i}\sim\:\mathcal{U}[-p,p]\:$characterizes with a tolerance level $\:p\in\:[0.1,\:0.2]$.

For each Monte Carlo iteration $\:k\in\:[1,N]$, an independent random realization $\:{\varvec{\theta\:}}^{\left(k\right)}$has been generated to construct the corresponding plant transfer function $\:{G}^{\left(k\right)}\left(s\right)$ the closed loop transfer function is defined for each controller $\:C\left(\text{s}\right)$, as in Eq. (30)

30 $$\:{L}^{\left(k\right)}\left(s\right)=C\left(s\right){G}^{\left(k\right)}\left(s\right),\:\:\:\:\:\:\:\:{T}^{\left(k\right)}\left(s\right)=\frac{{L}^{\left(k\right)}\left(s\right)}{1+{L}^{\left(k\right)}\left(s\right)},\:\:\:\:\:\:\:\:{S}^{\left(k\right)}\left(s\right)=\frac{1}{1+{L}^{\left(k\right)}\left(s\right)}.$$

In each iteration the stability is verified such that all the poles of $\:{T}^{\left(k\right)}\left(s\right)$ satisfy $\:\mathfrak{R}\left[\text{pole}\left({T}^{\left(k\right)}\left(s\right)\right)\right]<0$. The performances of rise time, settling time, overshoot percentage and peak sensitivity is evaluated. For each controller type, 2000 Monte Carlo simulations have been performed for the statistical estimation. The median value for each performance index is determined; and for the visualization the joint distribution of the of rise time $\:{t}_{r}$ and peak sensitivity $\:{M}_{s}$ have been visualized using a two-dimensional kernel density estimate $\:K\left(\cdot\right)$ to offer an intuitive performance density map to reflect the global robustness and $\:{h}_{x},{h}_{y}$ represent the bandwidth parameters, which are automatically selected according to Silverman’s rule to balance bias and variance in the estimated density; and is given by Eq. (31) as:

31 $$\:f({t}_{r},{M}_{s})=\frac{1}{N{h}_{x}{h}_{y}}\sum\:_{k=1}^{N}K\left(\frac{{t}_{r}-{t}_{r}^{\left(k\right)}}{{h}_{x}}\right)K\left(\frac{{M}_{s}-{M}_{s}^{\left(k\right)}}{{h}_{y}}\right)\:$$

### Plant 1: Integrating plant

To comprehensively evaluate the robustness of the proposed controller and contrast it with the existing ones, Monte Carlo simulation has been performed for parametrically uncertain plants, wherein the uncertain parameters were sampled within ± 20% of their nominal values, and the resulting distributions of $\:{t}_{r}$, $\:{t}_{s}$, $\:{M}_{p}$, and $\:{M}_{s}$ have been statistically analysed. Table 13 report the median value for the 2000 iterations for the distribution; it can be observed from the Table 13 that the proposed controller exhibited narrow performance distribution and maintained bounded sensitivity peaks across all trials, confirming its superior robustness and stability compared to the other methods.

**Table 13:** Median closed-loop performance comparison for integrating System.

	ZN – PID	GA-ISE-PID	QFT-Manual	QFT PSV	Proposed
Rise Time	0.246 s.	0.188 s.	0.012 s.	0.013 s.	1.160 s.
Settling Time	2.549 s.	3.791 s.	0.089 s.	0.047 s.	1.928 s.
Overshoot Percentage	36.99%	2.38%	13.22%	0%	0.23%
Peak Sensitivity	1.387	1.139	1.192	1.206	1.170

**Fig. 33:**
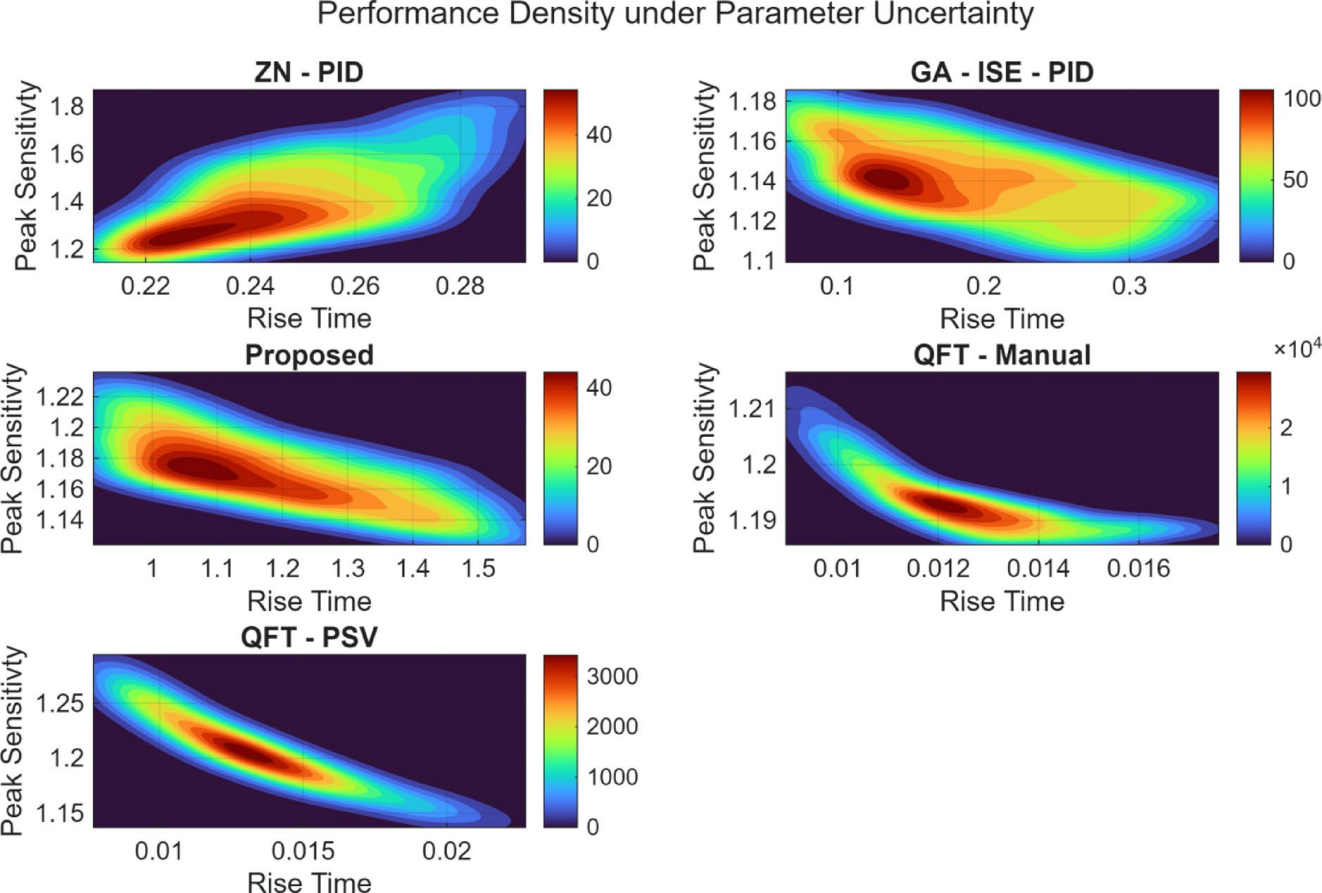
Performance density plots under plant parameter uncertainty for the integrating system.

Figure 33 shows the joint distribution of the rise time and the peak sensitivity obtained from the Monte Carlo simulation for 2000 uncertain plants; in each plot the colormap indicates the probability density; wherein the warmer colours represent the more frequent performance outcomes. In the case of Ziegler Nichols PID controller, a high spread is noticed indicating a poor robustness to plant variations. In case of GA-ISE PID controller offers a concentration of the responses and a moderate reduction in peak sensitivity and slightly varying rise times. Both QFT controllers, offer extremely narrow, high-density contours concentrated around low-rise times and moderate peak sensitivity, indicating strong closed loop performance and robustness. In case of the proposed controller, the density plot features a well clustered, moderately low-sensitivity region with slightly slower rise time but significantly improved robustness (low dispersion) and consistent transient performance. Thus, the controllers with tightly packed contours near low peak sensitivity values (e.g., proposed, QFT–PSV) achieve better tolerance to parametric uncertainty and stable closed-loop behavior across the uncertain parameter space.

### Plant 2: Position control of DC motor

**Fig. 34:**
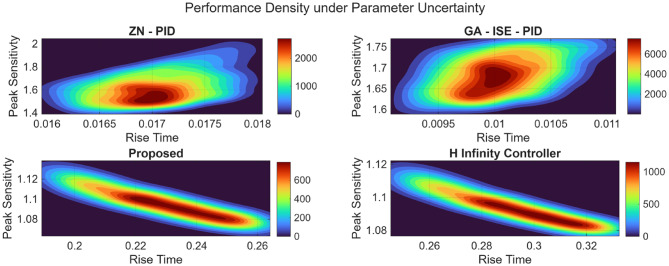
Performance density plots under plant parameter uncertainty for the position control of DC motor.

Similarly, the analysis is also done for the position control of DC motor, wherein the performance of the proposed controller is evaluated wing Monte Carlo simulations against the ZN-PID, GA-ISE-PID, and H_∞_ controllers under parametric uncertainties (± 10% of nominal values). Table 14 shows the median values for the performance metrics; and it is evident the proposed controller showcases superior stability and minimal performance variation. Figure 34 plots the performance density plots; which further establishes the robustness with a tightly clustered distribution of rise time and peak sensitivity.

**Table 14:** Median closed-loop performance comparison for position control of DC motor.

	ZN – PID	GA-ISE-PID	$\:{\mathcal{H}}_{\infty\:}$	Proposed
Rise Time	0.017 s.	0.010 s.	0.291 s.	0.223 s.
Settling Time	0.153 s.	0.059 s.	0.537 s.	0.413 s.
Overshoot Percentage	42.44%	25.39%	0%	0%
Peak Sensitivity	1.602	1.684	1.093	1.095

### Plant 3: NMP hydro power system

**Fig. 35:**
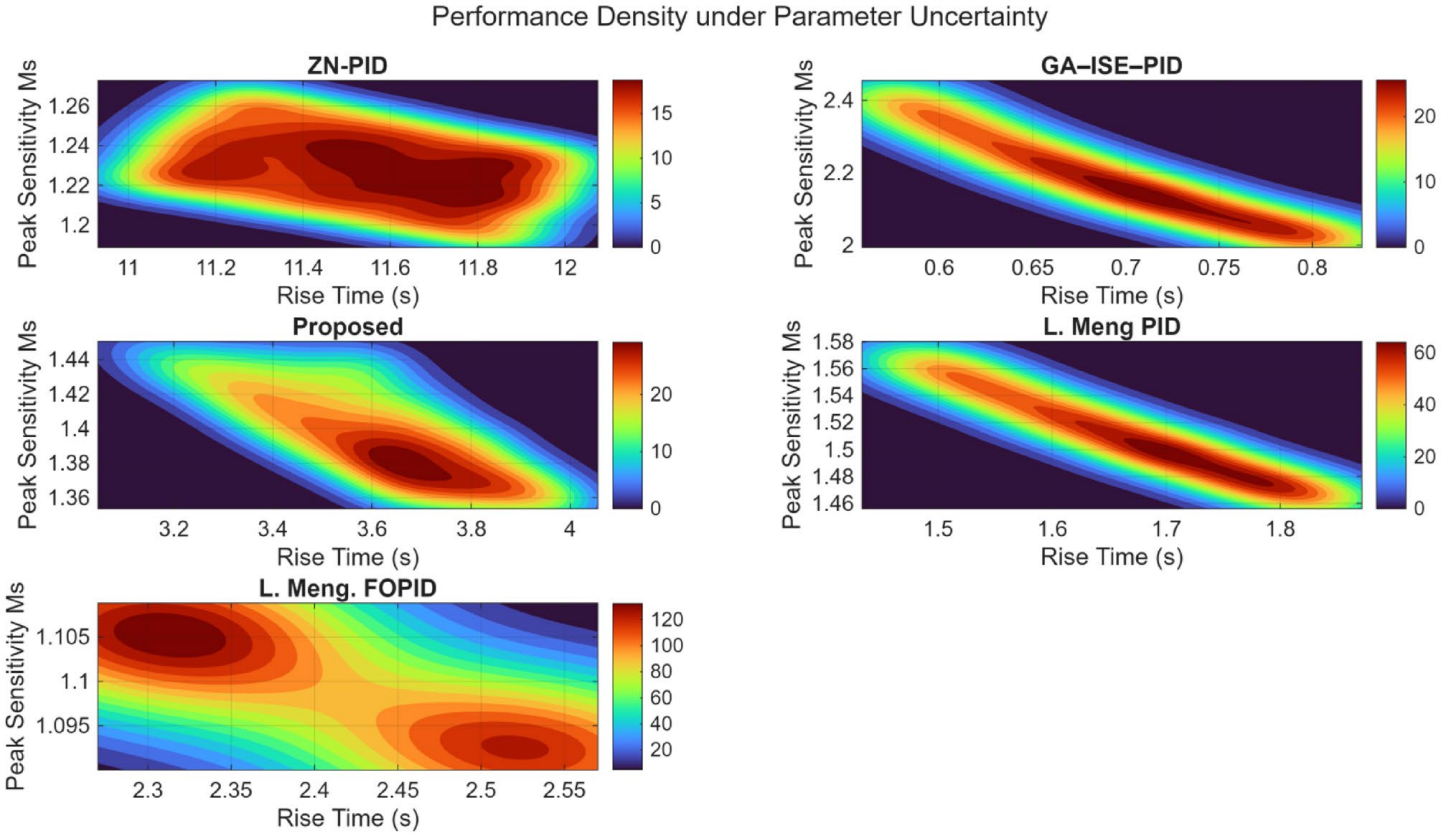
Performance density plots under plant parameter uncertainty for NMP hydro power system.

The robustness analysis is also performed for the NMP hydro power system using Monte Carlo simulations, wherein the proposed controller is compared with the ZN-PID, GA-ISE-PID, PID and FOPID controller proposed by L. Meng under parametric uncertainties. Table 15 shows the median values for the performance metrics; and it is evident the proposed controller showcases superior stability, indicating enhanced stability and minimal variation. Figure 35 plots the performance density plots; which further establishes the robustness with a tightly clustered distribution of rise time and peak sensitivity when compared with the wider spreads of ZN-PID and GA-ISE-PID, and the moderate performance of L. Meng PID and FOPID, thus establishing the superior performance offered by the proposed controller to parametric uncertainties.

**Table 15:** Median closed-loop performance comparison for NMP hydro power system.

	ZN – PID	GA-ISE-PID	L. Meng PID	L.Meng FOPID	Proposed
Rise Time	11.523 s.	0.688 s.	1.666 s.	2.370 s.	3.611 s.
Settling Time	36.095 s.	5.390 s.	11.757 s.	16.310 s.	9.852 s.
Overshoot Percentage	7.35%	11.11%	12.74%	17.28%	0%
Peak Sensitivity	1.288	2.172	1.508	1.10	1.394

### Plant 4: Coupled tank systems

**Fig. 36:**
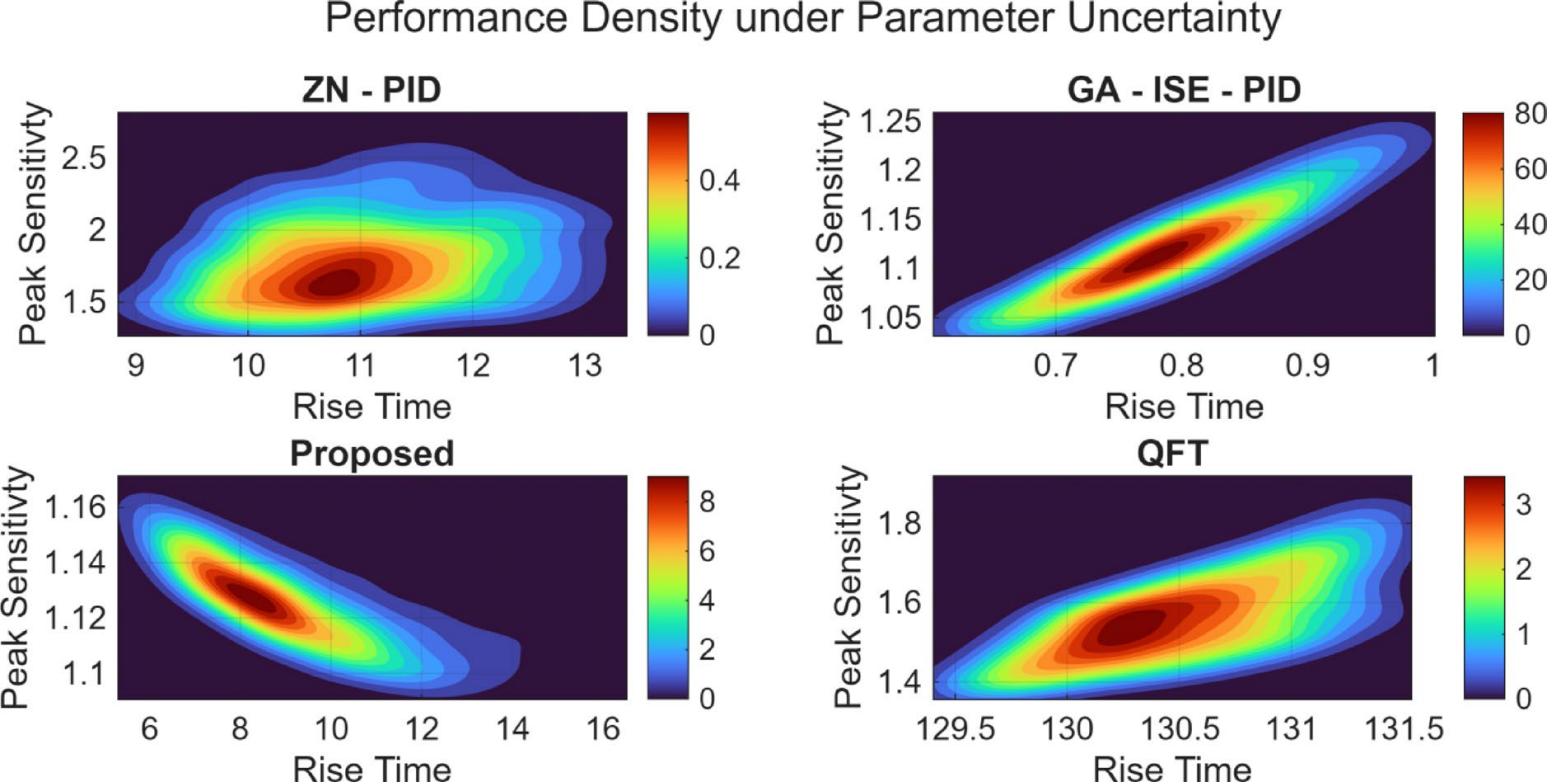
Performance density plots under plant parameter uncertainty for coupled tank system.

Finally, the Monte Carlo simulations are performed for coupled tank systems, and the proposed controller is compared with ZN-PID, GA-ISE-PID and the QFT controllers. Table 16 shows the median values for the performance metrics for all the controllers for parametrically uncertain plant; wherein the proposed controller offers superior stability and minimal variance. Figure 36 shows the density plots; and it can be observed that the proposed controller offers a very tight distribution for rise time and peak sensitivity, contrasting with the wider spreads of ZN-PID and GA-ISE-PID, and the slower but stable QFT, thus validating the enhanced robustness of the proposed controller in the presence of parametric uncertainties.

**Table 16:** Median closed-loop performance comparison for coupled tank system.

	ZN – PID	GA-ISE-PID	QFT	Proposed
Rise Time	10.916 s.	0.790 s.	130.505 s.	8.779 s.
Settling Time	133.316 s.	5.948 s.	236.127 s.	21.992 s.
Overshoot Percentage	44.33%	25.77%	0%	0%
Peak Sensitivity	1.743	1.117	1.551	1.124

**Fig. 37:**
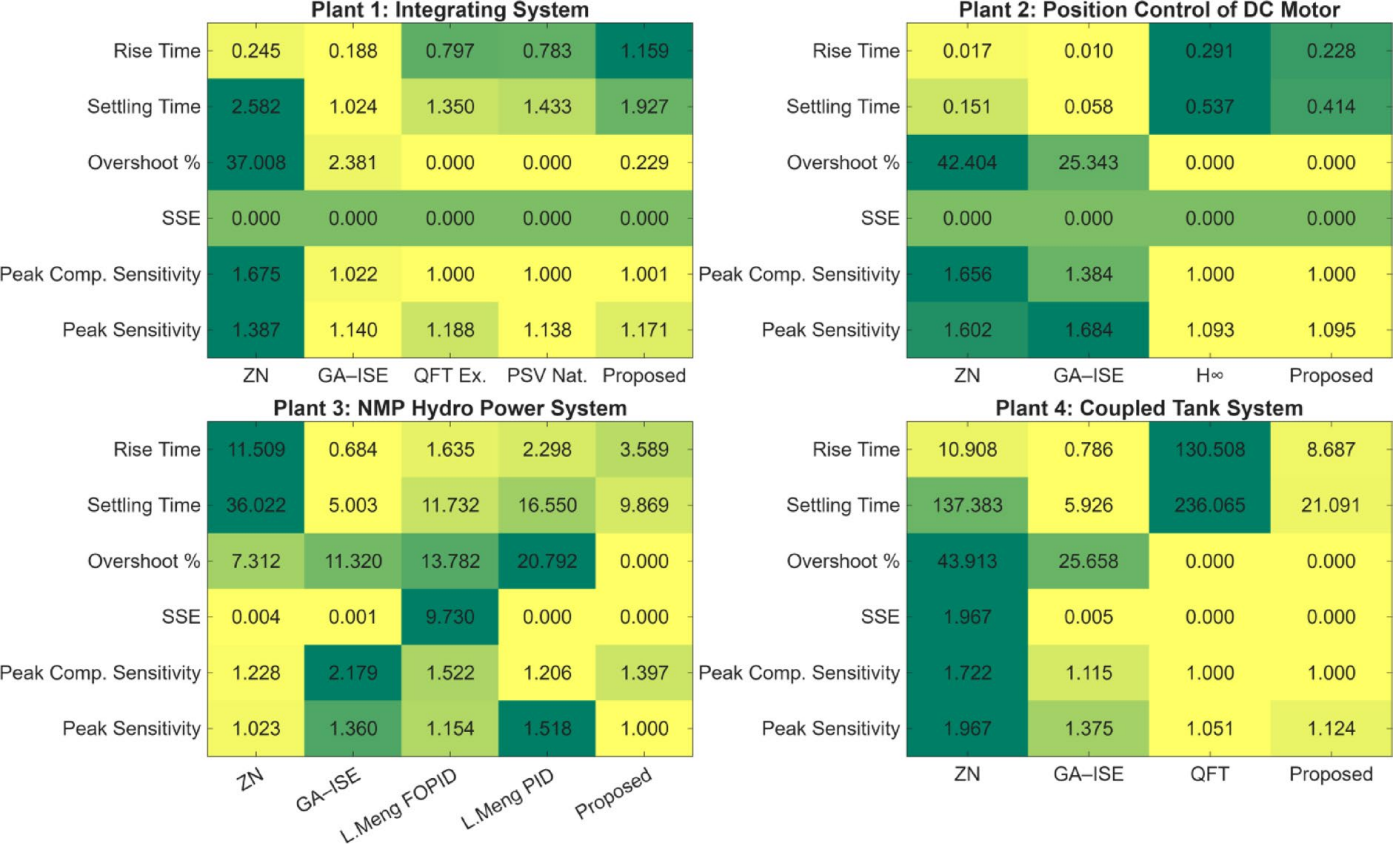
Comparison of control performance metrics for different plants with respect to the proposed method (lighter (*towards yellow*) values are better and darker (*towards dark green*) values are bad).

## Discussion

In Sect. 6, the quantitative analysis for closed loop response of synthesized controllers is presented for four considered plants. It can be observed that the controllers synthesized using the proposed multi-objective design offers better closed loop characteristics and has been validated by considering parametric uncertainties in Sect. 7. It can also be observed in the Sect. 6, that the synthesized controllers have better robustness characteristics in terms of the peak gain of sensitivity and complementary sensitivity functions; as well as the minimal control effort. Figure 33 shows plot for performance comparison between the ZN, GA-PID and proposed controllers for four plants for the indices reported in Sect. 6; and it is established that designed controller offers better robustness characteristics in comparison with the ZN-PID controllers and GA tuned PID controllers. In the Fig. 37, the heatmaps are used to visualize the normalized values of performance metric (lighter (yellow) the better); and is observed that proposed controllers offer a better response characterization.

## Conclusion

Most engineering problems are characterized by complex dynamics and multiple conflicting objectives, where ensuring optimal performance is critical for the satisfaction of specific performance criteria. As most of the real-world systems characterize complex dynamics, making the controller synthesis very challenging; and the use of classical control methods will not assure optimality and robustness. So, it is essential to model the controller synthesis as a multi-objective problem, wherein critical objectives can be considered for controller synthesis. In presented work, controller design is proposed as an MOOP and is solved using MOGA. The work is implemented for four systems like (a) integrating plants characterising integrals in the system transfer function; (b) position control of a DC Motor (integral plant), (c) NMP hydropower system and (d) coupled tank systems. The work also employs use of K-Means clustering to determine ideal solution from POS. Obtained results indicate superiority of proposed method when compared with existing controller; and compared results, establishes superiority of the proposed method over the conventional ones especially the robustness.

## Data Availability

No datasets were generated or analysed during the current study.
